# LRRC56 is an IFT cargo required for assembly of the distal dynein docking complex in *Trypanosoma brucei*

**DOI:** 10.1091/mbc.E23-11-0425

**Published:** 2024-07-11

**Authors:** Serge Bonnefoy, Aline Araujo Alves, Eloïse Bertiaux, Philippe Bastin

**Affiliations:** a Trypanosome Cell Biology Unit, Institut Pasteur, Université de Paris Cité, INSERM U1201, Paris, France; b Sorbonne Université, école doctorale complexité du vivant, ED 515, 7, quai Saint-Bernard, case 32, 75252 Paris Cedex 05, France; Arizona State University

## Abstract

Outer dynein arms (ODAs) are responsible for ciliary beating in eukaryotes. They are assembled in the cytoplasm and shipped by intraflagellar transport (IFT) before attachment to microtubule doublets via the docking complex. The LRRC56 protein has been proposed to contribute to ODAs maturation. Mutations or deletion of the *LRRC56* gene lead to reduced ciliary motility in all species investigated so far, but with variable impact on dynein arm presence. Here, we investigated the role of LRRC56 in the protist *Trypanosoma brucei,* where its absence results in distal loss of ODAs, mostly in growing flagella. We show that LRRC56 is a transient cargo of IFT trains during flagellum construction and surprisingly, is required for efficient attachment of a subset of docking complex proteins present in the distal portion of the organelle. This relation is interdependent since the knockdown of the distal docking complex prevents LRRC56’s association with the flagellum. Intriguingly, *lrrc56*^−^^/^^−^ cells display shorter flagella whose maturation is delayed. Inhibition of cell division compensates for the distal ODAs absence thanks to the redistribution of the proximal docking complex, restoring ODAs attachment but not the flagellum length phenotype. This work reveals an unexpected connection between LRRC56 and the docking complex.

## Abstract (executive summary)

This study addresses the unanswered question of why absence of LRRC56, a protein conserved in organisms with motile cilia assembled by intraflagellar transport (IFT), leads to loss of outer dynein arms (ODA) only in the distal portion of the Trypanosoma brucei flagellum.

The authors used functional analysis and live imaging to demonstrate that LRRC56 is an IFT cargo and that the interaction of LRRC56 with the distal docking complex is responsible for the absence of ODA.

This work revealed how LRRC56 is transported and unveiled an unexpected link with the dynein docking complex, which might explain some phenotypes in ciliopathy patients with LRRC56 mutations.

## INTRODUCTION

Ciliary motility is driven by the action of outer (ODA) and inner dynein arms, two large multi-protein complexes attached to the A-tubule of axonemal doublets. They are usually present on all nine doublet microtubules and distributed all along the axoneme. They are composed of a variable number of heavy, intermediate, and light chains (for a review on dynein composition, see King, 2016). Only the heavy chains have motor activity while intermediate and light chains are essential for the assembly and regulation of the complex. Dynein arms are assembled in the cytoplasm by a specific machinery itself composed of several subunits (Omran *et al*., 2008). They are then transported by intraflagellar transport (IFT) (Dai *et al*., 2018) and finally attached to the microtubule via the docking complex, a little structure itself fixed to the A-tubule (Qiu and Roy, 2022).

This machinery is remarkably well conserved throughout evolution and defects in any component of this sophisticated pathway perturbs dynein arm assembly, resulting in altered motility in all organisms investigated so far. This includes humans, where mutations in genes involved in dynein arm composition, assembly or transport cause primary ciliary dyskinesia (PCD, MIM: PS244400). This disease is characterized by impaired mucociliary clearance and increased susceptibility to respiratory infections, infertility, and *situs inversus* or other laterality disorders (Legendre *et al*., 2021).

Model organisms such as the green alga *Chlamydomonas* have been instrumental in deciphering the composition, assembly, and function of dynein arms (Silflow and Lefebvre, 2001). Identification of the first *PCD* gene that encodes the intermediate dynein chain 1 (DNA1 or IC78), a central component of the outer dynein arm, was guided by exhaustive structural and genetic knowledge in the green alga (Pennarun *et al*., 1999). Later on, it turned out that many components of the outer dynein arms and their assembly machinery were largely conserved in eukaryotes with motile cilia.

However, an unusual situation for outer dynein arms has been recently reported for the *LRRC56/ODA8* gene, which encodes a protein containing multiple leucine-rich repeats (leucine-rich repeat containing 56 protein) that is conserved in organisms with motile cilia assembled by IFT (Desai *et al*., 2015). Its impairment either by mutations in human patients or *Chlamydomonas*, or by double gene deletion in *Trypanosoma brucei* revealed a clear contribution to ciliary beating in all three species but surprisingly, the ultrastructural consequences were distinct (Bonnefoy *et al*., 2018). The *Chlamydomonas* null mutant is termed *oda8* and is characterized by the absence of outer dynein arms all along the axoneme (Kamiya, 1988; Desai *et al*., 2015). By contrast, a predicted null mutation in a human patient is not accompanied by visible structural defects in dynein arms of cilia from nasal biopsy samples, despite a dyskinetic beat pattern observed in air-liquid interface culture (Bonnefoy *et al*., 2018). Even more intriguingly, the knockout of *LRRC56* in *T. brucei* results in the formation of flagella where ODA is lacking from only the distal half of the axoneme, mostly in growing flagella (Bonnefoy *et al*., 2018).

In *Chlamydomonas*, the precise localization of the LRRC56 protein could not be determined by immunofluorescence but cell fractionation revealed that an HA-tagged version of the protein was present mostly in the cytoplasm and in the matrix of the flagellum (Desai *et al*., 2015) a distribution similar to that of IFT proteins (Ahmed *et al*., 2008). Using in vivo imaging, IFT-mediated transport of outer dynein arm components has been demonstrated upon expression of the intermediate chain IC2 (also called DNAI2) fused with a mNeonGreen (mNG) fluorescent reporter. In the *oda8/lrrc56* mutant, the frequency of ODA transport events is dramatically reduced, indicating defects either for entry in the flagellum or for association with IFT trains (Dai *et al*., 2018). Pull-down experiments in HEK293 cells showed that the human LRRC56 coimmunoprecipitates with IFT88, also suggesting an association with IFT (Bonnefoy *et al*., 2018). In trypanosomes, an YFP fusion protein expressed in situ from the *LRRC56* locus was found mainly at the distal portion of growing flagella, in an IFT-dependent manner and was solubilized upon detergent treatment. All these data indicate that LRRC56 interacts somehow with the IFT machinery in a wide range of organisms, but its precise functions remain to be deciphered, as well as why the absence of LRRC56 leads to unusually variable structural phenotypes between different organisms.

In an effort to explain the trypanosome situation where the *lrrc56* knockout mutant only lacks dynein arms in the distal portion of its axoneme (and yet mostly during elongation and not so much in mature flagella), we considered the peculiarities of flagellum composition and assembly in *T. brucei*. It is known that the axoneme is heterogeneous all along its length (Subota *et al*., 2014; Edwards *et al*., 2018; Billington *et al*., 2023). In particular, there are two distinct docking complexes: a proximal one that covers about the first half of the flagellum and a distal one, present only on the other half. Each is made of at least two subunits that contain coiled-coil domains (proximal: pDC1 and pDC2, distal: dDC1 and dDC2). pDC1 and dDC1 are related to the CCDC151/ODA3 family (Koutoulis *et al*., 1997; Hjeij *et al*., 2014; Jerber *et al*., 2014) while pDC2 and dDC2 are related to CCDC114/ODA1 (Takada *et al*., 2002; Knowles *et al*., 2013; Onoufriadis *et al*., 2013). In this context, we propose two hypotheses (not necessarily exclusive): 1) LRRC56 could only be required for the addition of dynein arms to the distal docking complex (dDC) and 2) LRRC56 could be required for efficient entry, transport or maturation of dynein arms in the trypanosome flagellum as observed in *Chlamydomonas* (Dai *et al*., 2018). In that second case, one could imagine that the rate of addition of dynein arms in *lrrc56*^−^*^/^*^−^ cells is too slow relative to that of elongation of the axoneme, hence limiting ODA presence to the proximal portion of the flagellum. This is supported by the fact that in the *lrrc56* knockout mutant, the mature flagella possess a higher proportion of dynein arms than the growing flagella (Bonnefoy *et al*., 2018).

We show here that LRRC56 is an IFT cargo and that a combination of the two proposed models can explain the distal absence of dynein arms in growing flagella and its partial compensation in mature ones but in a rather unexpected manner. In the absence of LRRC56, proteins of the dDC are present in very low concentration in the middle portion of the flagellum and absent from its distal part, hence explaining the lack of dynein arms in that region in growing flagella, and shedding light on an unexpected connection between LRRC56 and the docking complex. This appears interdependent as the knockdown of dDC1 or dDC2 prevents LRRC56 from reaching the flagellum. As flagella age over time, the proximal docking complex (pDC) occupies more extensive territories than in control cells, allowing further association of dynein arms that almost reach the distal end. These results answer the question as to why dynein arms were missing only in the distal portion of young flagella and how it is compensated as flagella mature.

## RESULTS

### mNG::LRRC56 is transported by IFT

**FIGURE 1:**
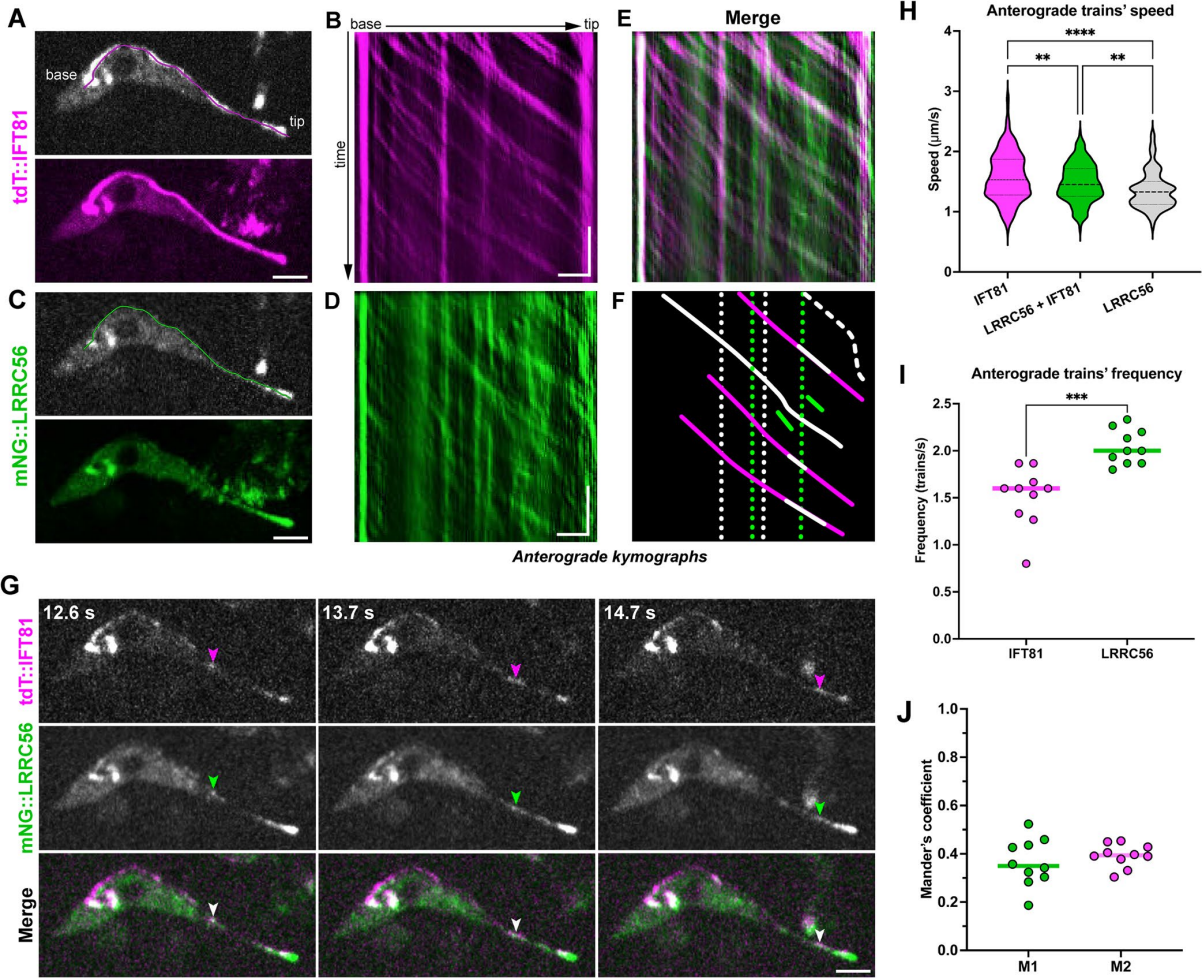
IFT transport of mNG::LRRC56 protein. (A–F) Representative analysis of a cell expressing tdT::IFT81 (magenta) and mNG::LRRC56 (green) following in situ tagging. (A) A still image of the red channel with tdT::IFT81 (top) shows this protein concentrates at the base of the flagellum and distributes along the length until the tip. In magenta, the region of interest used to extract the kymographs. Following the 15-s time lapse acquisition, the temporal projection of tdT::IFT81 (bottom) reveals its processive anterograde movement in the entire flagellar length. Scale bar: 3 μm. (B) The IFT81 anterograde kymograph of the cell in A shows this protein displacing from base to tip with a few arrested trains (vertical traces). Horizontal scale bar: 3 μm, vertical scale bar: 3 s. (C) A still image of the green channel (top) of the same cell in A showing mNG::LRRC56 (top). The same region of interest shown in green in A was used to extract the kymographs. The mNG::LRCC56 temporal projection (bottom) demonstrates that this protein concentrates at the distal portion of the flagellum. Scale bar: 3 μm. (D) The anterograde kymograph of the cell in C shows mNG::LRRC56 either as arrested material or as particles moving from the base toward the tip. Horizontal scale bar: 3 μm, vertical scale bar: 3 s. (E) Merge of IFT81 (B) and LRRC56 (D) anterograde kymographs reveals their partial colocalization. (F) A schematic summary of different events observed in E. The continuous white line indicates a particle containing both IFT81 and LRRC56 moving along the whole flagellum length. The dashed white line indicates a particle containing IFT81 and LRRC56 that alternates between moving and stalling. The dotted lines represent the arrested material of LRRC56 alone (green) or associated with IFT81 (white). Continuous lines show IFT81 particles (magenta) carrying LRRC56 for a short part of their trajectory (white lines). LRRC56 is also moving without being associated with IFT (green lines). (G) Still images of the same cell in A and C at the indicated timepoints confirm the displacement of an IFT particle (tdT::IFT81) transporting LRRC56 (arrowheads). See Supplemental Movie S1 for the full sequence. Images were processed to enhance contrast. Scale bar: 3 μm. (H) Quantification of anterograde trafficking speed of mNG::IFT81 (magenta) (*n* = 227 from 10 cells), mNG::LRRC56 particles moving together with IFT81 (green) (*n* = 88 from 10 cells) and mNG::LRRC56 particles moving alone (gray) (*n* = 219 from 10 cells) in monoflagellated cells. ^**^, *P*<0.01; ^****^, *P*<0.0001. (I) Quantification of the frequency of anterograde particles (*n* = 10 cells) shows LRRC56 particles are more abundant than IFT81. ^***^, *P* = 0.0004. (J) Manders’ colocalization analysis indicates a relatively low correlation between the trajectories of IFT81 and LRRC56.

Multiple data indicate that LRRC56 is somehow related to IFT (Desai *et al*., 2015; Bonnefoy *et al*., 2018). To evaluate whether LRRC56 is trafficking inside the flagellum and could be a cargo of IFT, we generated endogenous double-tagged mNG::LRRC56 and tdT::IFT81, a classic IFT marker, (Fort *et al*., 2016; Bertiaux *et al*., 2018a) in a wild-type background to image two color fluorescence simultaneously in live cells. Acquisitions were performed on monoflagellated cells displaying an mNG::LRRC56 fluorescent signal, therefore corresponding to cells that inherited a new flagellum after cytokinesis, as LRRC56 disappears during flagellum maturation as shown previously (Bonnefoy *et al*., 2018). Video observation showed the expected trafficking of the mNG::IFT81 protein, with generally processive anterograde movement from base to tip (Figure 1A; Supplemental Figure S1A; Supplemental Movies S1 and S2) exhibiting the expected speed and frequency (Figure 1, H and I). A few arrested trains were sometimes observed (Figure 1, B and E; Supplemental Figure S1B and S1E), and Supplemental Movies S1 and S2). These results agree with previously reported data for IFT protein trafficking in *T. brucei* (Huet *et al*., 2014; Fort *et al*., 2016; Bertiaux *et al*., 2018a). When observing the green channel, short fluorescent particles of mNG::LRRC56 displayed obvious anterograde movement (Figures 1D; Supplemental Figure S1D; Supplemental Movies S1 and S2). Retrograde traces were sometimes detected but were too weak to be analyzed with confidence, the analysis was therefore focused on anterograde movement. Frequent events of mNG::LRRC56 showing displacement from base to tip along the whole flagellum length were observed, as expected for a conventional IFT cargo (Figure 1F, continuous white line). However, many of these traces appeared less processive than tdT::IFT81 and were often restricted to the distal portion of the flagellum (see temporal projection in Figure 1C and Supplemental Figure S1C). Kymographs (space-time plots) analysis confirmed the presence of anterograde trafficking of tdT::IFT81 along the flagellum (Figure 1B; Supplemental Figure S1B) and revealed numerous traces of mNG::LRRC56 particle movement (Figure 1D; Supplemental Figure S1D). However, the pattern looked more complex and heterogenous than for mNG::IFT81, with a combination of arrested and moving particles, some showing alternation of movement with periods of stalling at various places along the distal portion of the flagellum (Figure 1D). The speed average of mNG::LRRC6 particles was similar to IFT trains, although significantly slower (Figure 1H, green violin), while their frequency was higher (Figure 1I). Merging the kymographs for tdT::IFT81 and mNG::LRRC56 revealed a frequent association between the two proteins (Figure 1, E–G; Supplemental Figure S1, E–G), providing the first evidence of LRRC56 transport as cargo by the IFT machinery. When IFT trains were arrested, mNG::LRRC56 was also frequently associated with them (dotted white lines, Figure 1F). However, the IFT and LRRC56 association appeared transitory, with LRRC56 being “dropped off” (see, e.g., Supplemental Figure S1F, dashed green line) and remaining stationary without further colocalization with IFT (Supplemental Figure S1F, dotted green lines). Nevertheless, these stationary LRRC56 particles can be “picked up” by an IFT train (see Supplemental Figure S1F, dotted green line). Intriguingly, many moving mNG::LRRC56 particles not associated with IFT trains were also observed (green lines, Figure 1F) and showed lower speeds (Figure 1H, gray violin). The Manders’ overlap coefficient demonstrated that 36% of mNG::LRRC56 moving particles were colocalizing with tdT::IFT81 and that 40% of IFT trains were colocalizing with mNG::LRRC56 (Figure 1J). This shows that only a fraction of the mNG::LRRC56 flagellar pool is transported as cargo of the IFT system.

**Figure:** Movie S1

**Figure:** Movie S2

### Partial absence of the dDC in *lrrc56* ^−/−^ cells

**FIGURE 2:**
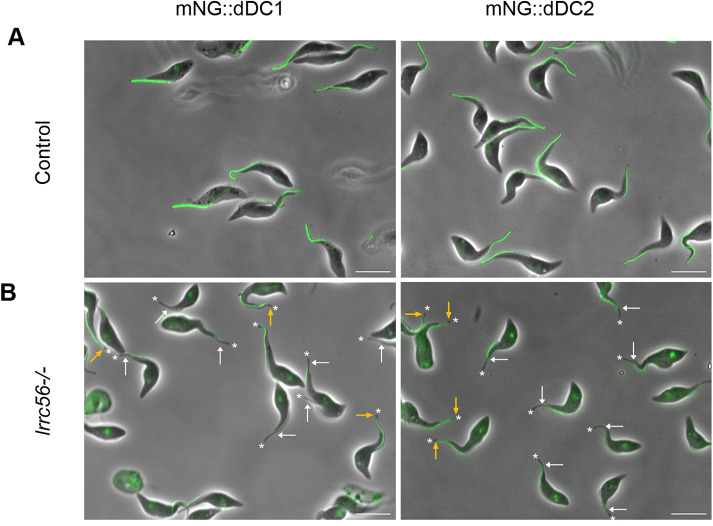
The dDC proteins are missing from the distal part of the flagellum in *lrrc56*^−/−^ cells. In situ tagging of dDC1 or dDC2 with mNG was performed in wild-type (A) or *lrrc56*^−/−^ cells (B). In control cells, both mNG::dDC1 and mNG::dDC2 are only found in the distal half portion of the axoneme as expected (A). By contrast, in many *lrrc56*^−/−^ cells, mNG::dDC1 or mNG::dDC2 fluorescence signals are missing from the very distal portion of the axoneme (B). The white arrows show the end of the fluorescent signal while the white asterisks show the tip of the flagellum. Distal absence of mNG::dDC2 signal in live *lrrc56*^−/−^ cell line is observed in about half of the cells (indicated with white arrows) while the other cells harbor complete or nearly complete dDC2 flagellar signal except for the tip (yellow arrows). Asterisks show the end of the flagellum. A faint signal is also observed between the nucleus and the kinetoplast of tagged mNG::dDC2 cells, possibly reflecting some lysosome degradation. Scale bars: 10 µm.

Having found out that LRRC56 was an IFT cargo, we next sought to explain why it was required for the addition of ODA only to the distal part of the flagellum. The first hypothesis consists of a differential dependence along the length of the flagellum. One strong candidate for this is the docking complex since it is made of a proximal and a distal one in trypanosomes (Edwards *et al*., 2018), but not in *Chlamydomonas* where its composition is homogeneous from base to tip of the axoneme (Owa *et al*., 2014). We first examined whether LRRC56 absence had an impact on the flagellar localization of the dDC members dDC1 and dDC2. Each of the proteins was endogenously tagged with the mNG fluorescent marker in wild-type and *lrrc56*^−/−^ cells. In control cells, live fluorescence imaging revealed that both mNG::dDC1 and mNG::dDC2 distribute to the distal part of the flagella, no matter the phase of construction (Figure 2A), exactly as described (Edwards *et al*., 2018). By contrast, the signal intensity for mNG::dDC1 and mNG::dDC2 was significantly reduced and both proteins often failed to reach the tip of the flagellum in *lrrc56*^−/−^ cells (Figure 2B, white arrows). This pattern is consistent with the absence of outer dynein arms at the distal end of the flagellum reported in *lrrc56*^−/−^ cells (Bonnefoy *et al*., 2018). This result suggests that the cause of the absence of ODA at the distal end is the lack of the dDC.

**FIGURE 3:**
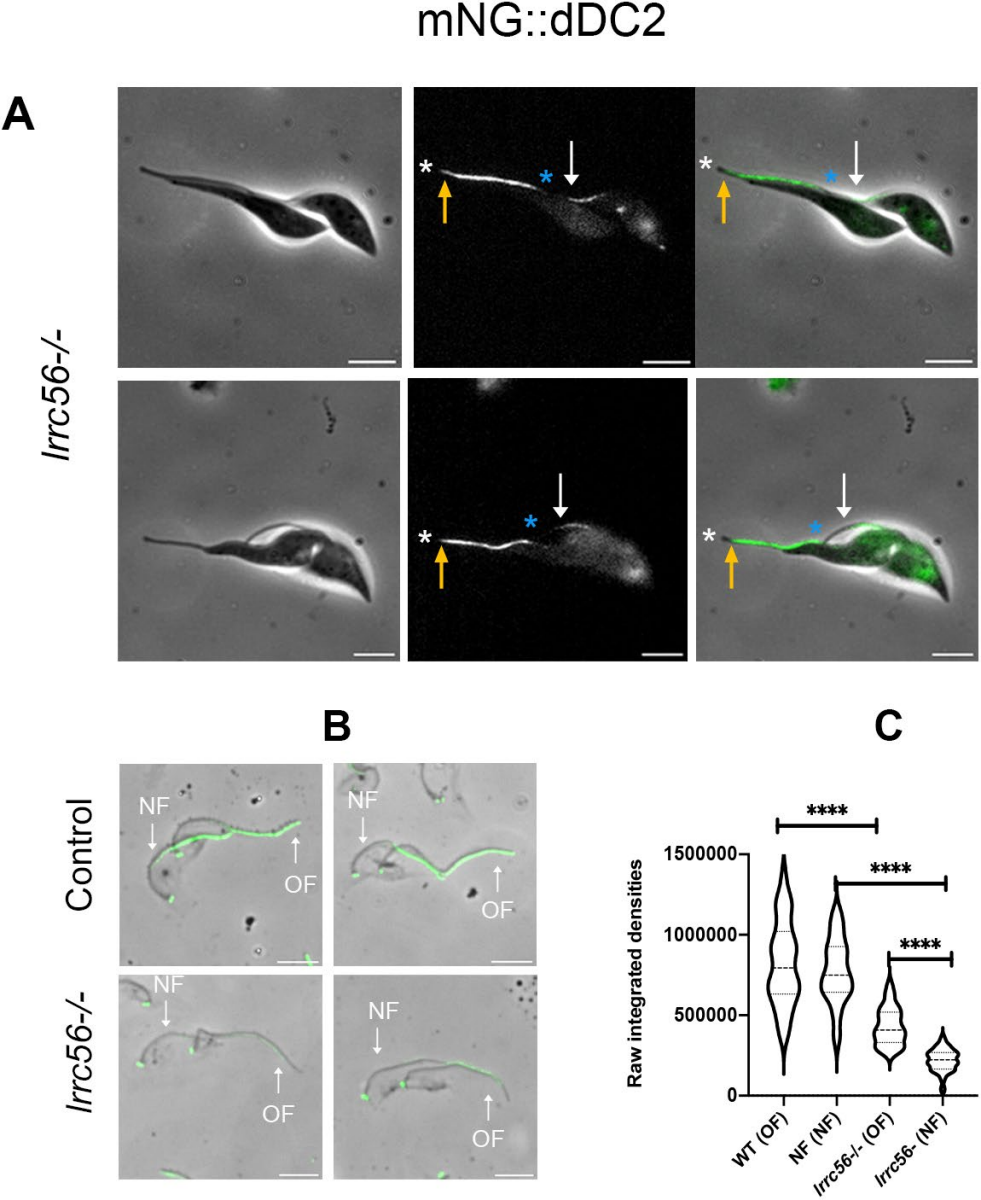
Reduced presence of mNG::dDC2 in *lrrc56*^−/−^ cells is mostly encountered in flagella under construction. (A) In live cells with two flagella about to divide, a nearly complete mNG::dDC2 signal (end of the signal, yellow arrows) is observed in the mature flagellum while in the new flagellum, the signal for mNG::dDC2 (end of the signal, white arrows) is limited to a short, central portion. The white and blue asterisks show the tip of the mature flagellum (OF) and the new flagellum (NF), respectively. Scale bars: 5 µm. (B) In detergent-extracted *lrrc56*^−/−^ cytoskeletons analysed by IFA using an anti-mNG antibody, the absence of mNG::dDC2 is observed at the distal tip of both the old and the new flagellum (white arrows), with reduced signal intensity compared with controls. Images are normalized. Scale bar: 5 µm. (C) Integrated density quantification using ImageJ of the total mNG::DC2 signal intensity along flagella in control and *lrrc56*^−/−^ detergent-treated cells revealed a 2-fold decrease in the new flagellum (NF) compared with the old flagellum (OF). 29 cells at 2K2N stage were analysed for both wild-type and *lrrc56*^−/−^. *P*-value for each paired data variation was <0.0001 (****).

Curiously, this phenotype of the absence of dDC1/2 proteins in *lrrc56*^−/−^ trypanosomes is observed in only about half of cells (Figure 2B, white arrows) while the others harbor complete or nearly complete dDC1/2 staining (Figure 2B, yellow arrows). To understand the reasons for this heterogeneity, we looked for a possible relationship between flagellum age and maturation (Bertiaux *et al*., 2018b). Trypanosomes that are about to divide possess two flagella, the new one that is under construction and the old one that has been assembled at least one generation before (Sherwin and Gull, 1989). While the mNG::dDC2 signal almost reached the tip of the old flagellum in biflagellated cells (Figure 3A, yellow arrows), the protein is barely detected in the new flagellum, where it fails to reach the distal extremity (Figure 3A, white arrows).

To evaluate whether the mNG::dDC2 protein was correctly attached to the axoneme, cytoskeletons were extracted with detergent, a procedure that removes all soluble material (Robinson *et al*., 1991). In control cells, the mNG::dDC2 protein was tightly associated with the axoneme at its expected distal location in both old and new flagella (Figure 3B, top panels). Some mNG::dDC2 detected by immunofluorescence assay (IFA) in *lrrc56*^−^*^/^*^−^ cells remained associated with the cytoskeleton (Figure 3B, bottom panels), showing that at least a part of it was properly anchored to microtubules. Because this signal looked weaker compared with controls, its intensity was quantified. No significant differences were seen between old and new flagella of the control cell line. By contrast, a 2-fold reduction of the mNG::dDC2 signal was observed in old flagella of *lrrc56*^−^*^/^*^−^ cells compared with controls and a 4-fold reduction in new flagella (Figure 3C).

### Redistribution of the pDC proteins in *lrrc56* ^−/−^ cells

**FIGURE 4:**
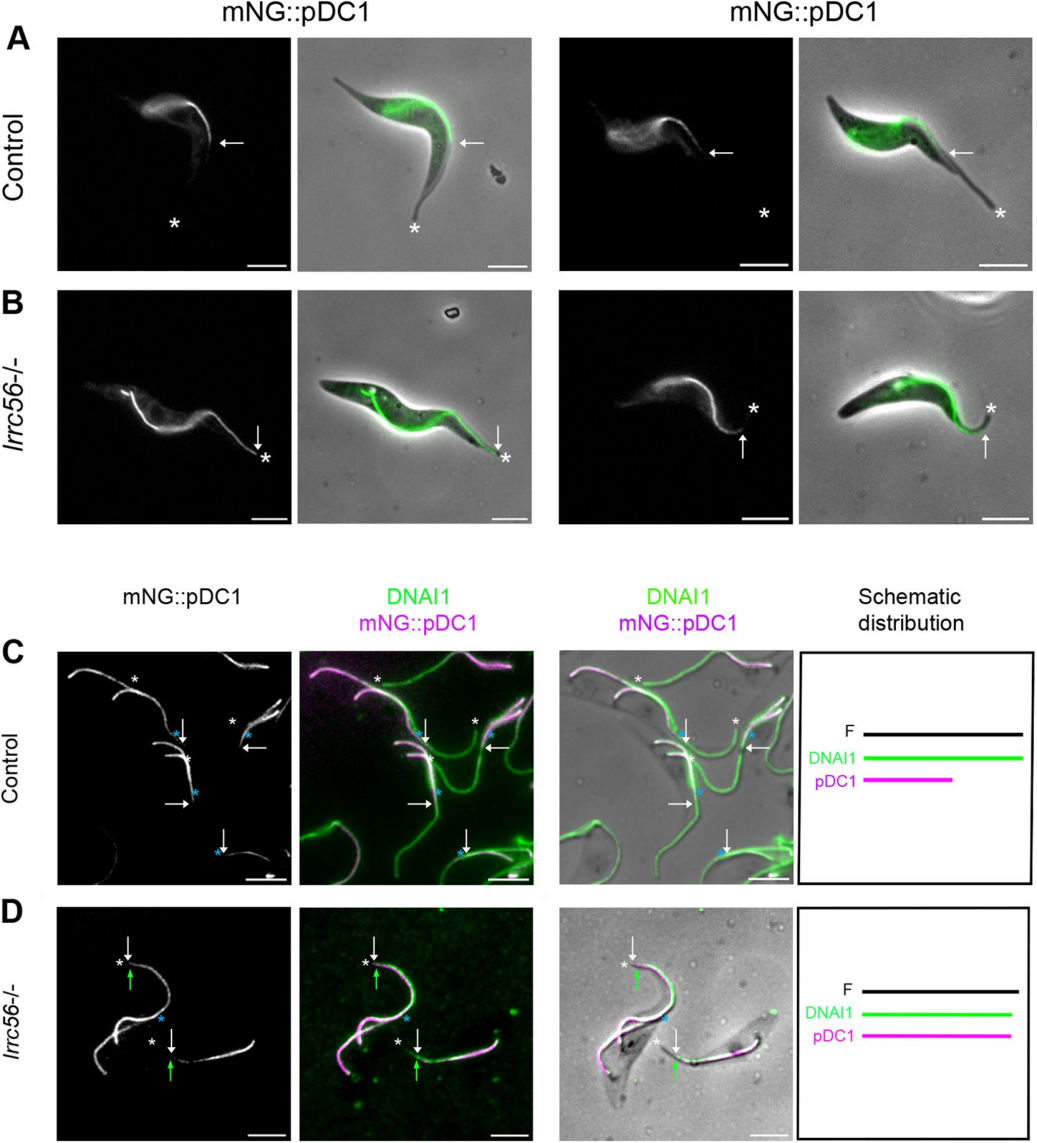
The pDC covers a much longer portion of the flagellum in *lrrc56*^−/−^ cells and colocalizes with outer dynein arms. (A, B) In situ tagging of pDC1 with mNG in wild-type (A) or *lrrc56*^−^*^/^*^−^ cells (B). The mNG::pDC1 signal (green) was merged with the phase contrast image. mNG::pDC1 signal alone is shown at the left of each merged image. The pDC protein is distributed in the proximal portion of the axoneme (about half-length) in the control situation (the white arrows show the end of the fluorescent signal), as expected (A). The distal end of the flagella is shown by a white asterisk. By contrast, the mNG::pDC1 signal extends toward the distal region of the flagellum in *lrrc56*^−/−^ cells (white arrows) (B), sometimes even reaching the distal tip (left panel). Scale bars: 5 µm. (C–D) Cytoskeletons of control (C) or *lrrc56*^−/−^ cells (D). Cytoskeletons of control (C) or *lrrc56*^−/−^ cells (D) expressing mNG::pDC1 (white in first panel, magenta in other panels) or the outer dynein arm component DNAI1 (green, central and right panels). (D) In *lrrc56*^−/−^ cells, the mNG::pDC1 signal extends toward the distal region of *lrrc56*^−/−^ flagella (white arrow) correlating to the extent of DNAI1 localization (green). The end of DNAI1 localization is shown by a magenta arrow in the *lrrc56*^−/−^ axoneme. The white asterisks show the end of the mature flagellum in the biflagellated cell (left) and the monoflagellated one (right), while the blue one indicates the tip of the new flagellum. Scale bars: 5 µm. Schematic representation of the dynein arm intermediate chain 1 (DNAI1, green) and pDC1 (magenta) distribution in control and *lrrc56*^−/−^ mature flagellum (F) is shown at the right of panel C and D.

When the dDC is absent upon inducible RNAi knockdown of any of its components, members of the proximal complex (mNG::pDC1 and mNG::pDC2) remain proximal but both components spread toward the distal end, albeit not totally (Edwards *et al*., 2018). We therefore, wondered whether their localization was also affected in *lrrc56*^−/−^ cells given the partial depletion of dDC proteins. We have explored this using control or *lrrc56*^−/−^ cells expressing mNG::pDC1 following in situ tagging. In control cells, we observed the previously described proximal localization of pDC1 in the first half of the flagellum as expected (Figure 4A). By contrast, the mNG::pDC1 signal extended on much longer portions of the flagellum in *lrrc56*^−/−^ cells, sometimes even reaching the tip of the organelle (Figure 4B).

### Dynein arms distribution relies on the pDC in *lrrc56* ^−/−^ cells

**FIGURE 5:**
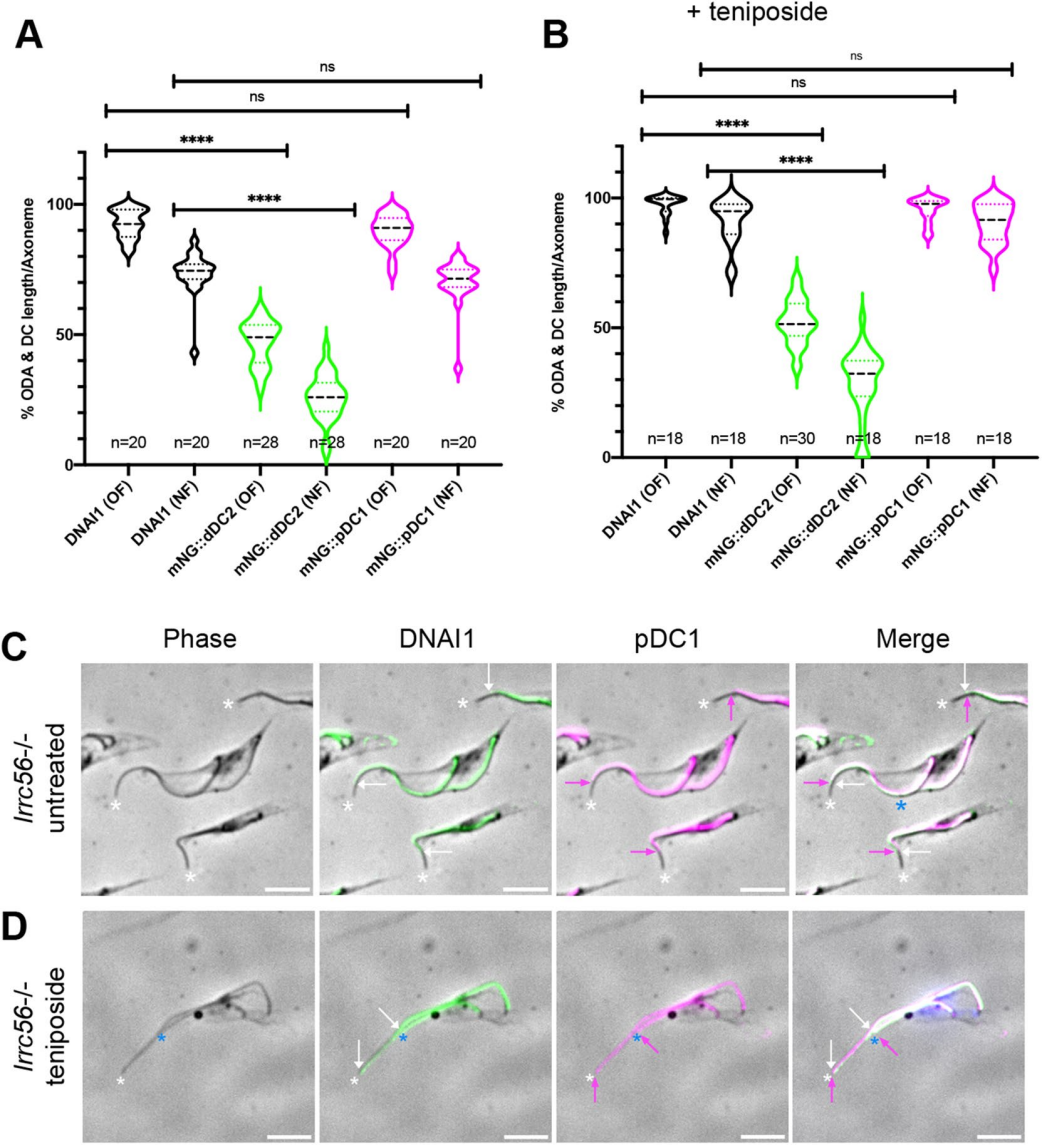
Inhibition of cell division restores most of the outer dynein arms despite the absence of LRRC56. (A) Distribution of the outer dynein arms (ODA labeled with DNAI1, black violins), distal (mNG::dDC2, green violins) and proximal (mNG::pDC1, magenta violins) docking complexes in old (OF) and new (NF) flagella in *lrrc56*^−/−^ cells, showing a correlation between DNAI1 axonemal location and pDC1 redistribution along the *lrrc56*^−/−^ axoneme. The ratio between the length occupied by the indicated protein and the length of the axoneme is shown in percentage. The violin plots represent the percentage of the length of the flagellum that is covered by ODA (DNAI1), distal (dDC2) and proximal (pDC1) docking complex proteins, respectively. The axonemal length was measured using the phase contrast image of the flagellum. *P*-value for each paired data variation was <0.0001 (****). (B) Teniposide-treated *lrrc56*^−^*^/^*^−^ mNG::dDC2 and *lrrc56*^−^*^/^*^−^ mNG::pDC1 cytoskeletons were stained with the anti-DNAI1 antibody as a marker of outer dynein arms and both DNAI1 axonemal distribution and mNG::DC fluorescent signals remaining after fixation were measured. (C–D) IFA images of cytoskeletons extracted from *lrrc56*^−^*^/^*^−^ mNG::pDC1 cells untreated (C) or teniposide treated for 18 h (D) stained for DNAI1 (green) or mNG::pDC1 (magenta). Distal tips of flagella are shown by white asterisks (monoflagellated or mature flagellum) or blue asterisks (new flagellum). The end of DNAI1 localization is shown by a white arrow in the *lrrc56*^−/−^ axoneme and the end of the mNG::pDC1signal by a magenta arrow. In *lrrc56*^−/−^ cells treated with teniposide (D), the mNG::pDC1 and DNAI1 signals colocalize and extend toward the distal tip of both the mature and the new flagellum. Scale bars: 5 µm.

To explore whether this redistribution was associated with outer dynein arm docking, control or *lrrc56*^−/−^ trypanosomes expressing mNG::pDC1 were stained with the anti-DNAI1 antibody (dynein arm intermediate chain 1, also known as IC78) (Branche *et al*., 2006) that recognizes an essential structural component of the ODA. In contrast to control cells (Figure 4C), the signals for DNAI1 and mNG::pDC1 almost perfectly overlap in mature and growing flagella in the absence of LRRC56 (Figure 4D). This redistribution of the pDC along the axoneme could be explained if components of the pDC were diffusing slowly from the base to the tip and filling progressively the unoccupied distal docking sites, explaining the discrepancies between distal loss of dDCs proteins and more extended axonemal association of ODAs mostly on the old flagellum*.* To quantify these results, the distribution of outer dynein arms, dDC, and pDC were measured using as reporters DNAI1, mNG::dDC2 and mNG::pDC1, respectively (Figure 5A). The mNG::dDC2 signal covered only 47% of the length of the old flagellum (Figure 5A) and dropped to barely 26% in new flagella in *lrrc56*^−/−^ cells (Figure 5A, green violins). By contrast, signals for the proximal docking component expanded to 72% and close to 90% of the total length in new and old flagella, respectively (Figure 5A, magenta violins), a profile that matches closely the distribution of the outer dynein arm marker DNAI1 (Figure 5A, black violins).

### Dynein arm assembly can be restored in *lrrc56* ^−^ *^/^* ^−^ cells

The above results show that the absence of dynein arms at the distal part of growing flagella is related to the molecular heterogeneity of the flagellum along its length, with interdependence between LRRC56 and the proteins of the dDC. However, the fact that ODA absence is less pronounced in mature flagella indicates that axoneme elongation and ODA addition might not take place at the same rate in *lrrc56*^−^*^/^*^−^ cells, with possibly further addition of ODA after cell division. Because further ODA attachment appears due to the presence of the pDC, this observation could be explained if the pDC components were diffusing slowly from the base toward the tip of the flagellum. In this model, older flagella would bind pDC components on longer portions and therefore would recover more ODAs in a base-to-tip gradient.

To evaluate this possibility, we took advantage of the fact that procyclic trypanosomes assemble their new flagellum in a strictly controlled cell cycle manner, undergoing cytokinesis when the ratio between new and old flagella is only ∼80%, with the rest of the construction taking place after cell division (Sherwin and Gull, 1989; Robinson *et al*., 1991; Farr and Gull, 2012; Bertiaux *et al*., 2018b) (Supplemental Figure S3A, black violins). We have chemically blocked cell division in *lrrc56*^−^*^/^*^−^ cells and control cells expressing mNG::dDC2, mNG::pDC1 or mNG::pDC1 with a 20 h incubation in the presence of 200 µM teniposide, an inhibitor of topoisomerase II that interferes with mitochondrial DNA segregation, but not basal body duplication nor flagellum elongation (Robinson *et al*., 1991). This inhibition of cell division allows the completion of the assembly of the new flagellum that reaches the same length as the old one in control cells (Bertiaux *et al*., 2018b; Atkins *et al*., 2021) (Supplemental Figure S3B, black violins). Detergent-extracted cytoskeletons were analysed by IFA using the anti-DNAI1 antibody. Flagellum length was measured and the ratio between old and new flagella was calculated (Figure 5, B and C; Supplemental Figures S3B and S4, A–C). In wild-type cells, new flagella have a length at ∼85% of their old counterpart (Supplemental Figure S3B) and that value climbs close to 100% when cell division is inhibited (Supplemental Figure S3B, black violins), meaning that the length of the new flagellum has reached the length of the old one as reported previously (Bertiaux *et al*., 2018b). We noted previously that flagella of *lrrc56*^−^*^/^*^−^ cells were shorter than control cells (Bonnefoy *et al*., 2018) (Supplemental Figure S3A, blue violins). Surprisingly, blockage of cell division barely modified the relative length of the new flagellum in *lrrc56*^−^*^/^*^−^ cells that moved from 77% to only 81% of the old flagellum length (Supplemental Figure S3B, blue violins), a difference that is not statistically significant (*P* = 0.102). By contrast, the presence of dynein arms along old and new flagella was significantly changed in teniposide-treated *lrrc56*^−^*^/^*^−^ cells, with dynein arms covering almost the entire length of the axoneme (Figure 5B, black violins, see Figure 5C and Supplemental Figure S4 for representative images). This was especially spectacular for new flagella where DNAI1 covers 91% of the length of the axoneme compared with a bit more than 70% in untreated *lrrc56*^−^*^/^*^−^ cells (compare black violins between Figure 5A and B). Similarly, the distribution of pDC1 significantly changed in teniposide-treated cells (Figure 5, A and B, magenta violins), becoming almost identical to the distribution of the outer dynein arms (Figure 5C). This result could be explained if the pDC components diffuse slowly from base to tip: the incubation for 20 h in the presence of teniposide prevented cells from dividing, giving sufficient time to pDC components to occupy the sites left vacant by the dDC in a base-to-tip gradient manner. It should be noted that the dDC also seems to expand further during flagellum maturation, as seen with longer portions of signal for mNG::dDC2 in old flagella compared with new flagella (Supplemental Figure S4).

### RNAi knockdown of *dDC2* impairs LRRC56 flagellum localization

The dDC therefore relies on LRRC56 for efficient association with the distal part of the flagellum. To investigate for a reciprocal relationship, we generated inducible RNAi cell lines targeting the *dDC2* gene and expressing mNG::dDC2, mNG::dDC1 or mNG::LRRC56 proteins as reporters. The efficiency of *dDC2* silencing was demonstrated following the endogenous expression of mNG::dDC2. Almost complete loss of the signal (Supplemental Figure S2) was observed upon RNAi induction. In agreement with the prediction that in *Chlamydomonas reinhardtii* the docking complex proteins DC1 and DC2 interact to form a heterodimer (Koutoulis *et al*., 1997; Takada *et al*., 2002) dramatic loss of the flagellar mNG::dDC1 signal was observed after *dDC2* silencing (Supplemental Figure S2) (Edwards *et al*., 2018).

**FIGURE 6:**
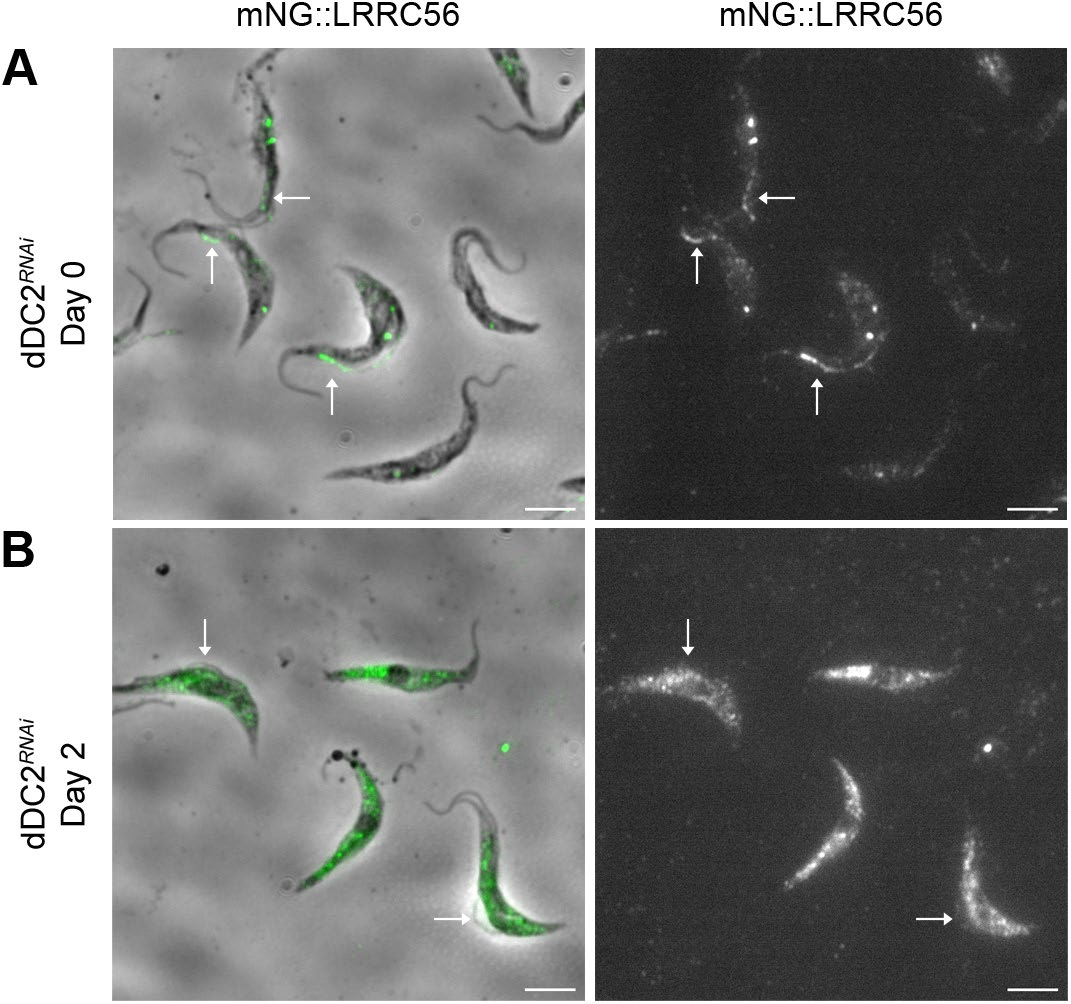
Loss of LRRC56 axonemal localization in the absence of the dDC. Live *dDC2^RNAi^* cells expressing the mNG::LRRC56 protein were observed in control or RNAi-induced conditions. In noninduced cells, the mNG::LRRC56 protein is localized in the distal half portion of growing flagella (A). Following 2 d of *dDC2* RNAi induction (B), the mNG::LRRC56 signal is no longer detected in new flagella but the protein is found in cytoplasmic accumulations (B). The images are normalized using ImageJ according to minimum and maximum pixel values. New flagella are indicated by white arrows. Scale bars: 5 µm.

Having confirmed the efficiency of dDC2 knockdown, we examined its impact on the presence and localization of LRRC56 upon in situ tagging with mNG. In the absence of RNAi induction, the mNG::LRRC56 protein was found at the distal part of flagella undergoing assembly (Figure 6A), as previously reported for YFP::LRRC56 (Bonnefoy *et al*., 2018). Strikingly, the mNG::LRRC56 flagellar signal was lost in induced *dDC2^RNAi^* cells and the protein was found accumulating in the cytoplasm (Figure 6B) as observed for mNG::dDC1 (Supplemental Figure S2). This result reveals that LRRC56 localization in the flagellum is dependent upon the dDC proteins. This phenotype is more pronounced compared with the reduction (but not disappearance) of dDC proteins in the absence of LRRC56 (Figures 2 and 3).

## DISCUSSION

Here, we provide the first direct evidence that LRRC56 traffics in the trypanosome flagellum with similar speed as reported for anterograde IFT. A significant amount of LRRC56 remains stalled with transient episodes of anterograde or retrograde transport, suggesting that LRRC56 would be a transient cargo of IFT trains. Such sequential anterograde progression has also been reported for IFT cargo proteins in *Chlamydomonas* (Wren *et al*., 2013; Craft *et al*., 2015; Dai *et al*., 2018). This observation explains the IFT-dependent localization reported previously (Bonnefoy *et al*., 2018) and enhances the link between LRRC56 and IFT. However, LRRC56 is able to move within the flagellum independently from IFT. The pattern and relatively fast speed of this movement are unlikely to correspond to diffusion, and the precise mechanisms of these transport events of LRRC56, remain an open question. Apart from the kinesin-2 molecular motors responsible for anterograde transport (Bertiaux *et al*., 2018b), more than 40 kinesin-like proteins are encoded by the *T. brucei* genome (Berriman *et al*., 2005), several of them being located in the flagellum (Subota *et al*., 2014; Billington *et al*., 2023) that might account for the independent transport of LRRC56. Phylogenetic distribution of *LRRC56* gene orthologs demonstrated that it is present only in organisms with motile cilia relying on IFT for assembly (Desai *et al*., 2015). Expression of the *Drosophila LRRC56* is only observed in chordotonal neurons, the only ones to possess motile cilia assembled by IFT in this organism. Accordingly, it is not detected in the testis where sperm flagellum assembly does not require IFT (Zur Lage *et al*., 2019). Moreover, biochemical analysis has shown that the *Chlamydomonas* LRRC56 protein shares a distribution profile similar to IFT (Desai *et al*., 2015). Finally, pull-down experiments indicate that the human LRRC56 interacts with the IFT88 protein following overexpression in HEK293 cells (Bonnefoy *et al*., 2018).

A second major finding of this study is the unexpected tight interdependence between LRRC56 and the dDC. Depletion of dDC components abolishes LRRC56 presence in the flagellum while knock out of LRRC56 leads to a severe reduction of dDC proteins, hence explaining the absence of ODA only in the distal portion of the flagellum. The apparent more severe impact of the knockdown of dDC proteins on LRRC56 has to be put in perspective with the significantly different amounts of each protein. LRRC56 was not detected by mass spectrometry of purified flagella, while a large number of peptides derived from dDC components were found: 20 and 14 peptides for dDC1 or 18 and nine for dDC2 in studies of detergent-extracted flagella or intact flagella, respectively (Broadhead *et al*., 2006; Subota *et al*., 2014). Moreover, the progressive rescue of ODA docking by the pDC over time solves the problem of the presence of dynein arms on longer portions of flagella as they mature. To explain how LRRC56 could contribute to the proper location of the dDC in the flagellum, we propose that it could function as an IFT adapter protein. Several of these have been reported to facilitate interactions between IFT and candidate cargo molecules (review in Lechtreck, 2022). Such a function could act at the level of entry of dDC components (allowing their passage through the transition zone) or of their transport toward the distal end of the flagellum. Another function could be the unloading of the dDC and perhaps its proper attachment to microtubules.

Edwards *et al.*, 2018 have proposed that retrograde transport of pDCs generates a concentration gradient in which the higher-affinity pDC is restricted to the proximal axoneme, while the lower-affinity and unbound dDC would diffuse throughout the flagellum and fill the remaining axoneme binding sites. In the absence of distal complex proteins, those of the pDC progressively occupy the space left vacant and ensure proper attachment of the dynein arms. Nevertheless, they are restricted to ∼75% of the flagellum length (Edwards *et al*., 2018). Here, in the absence of LRRC56 protein, pDC components can reach more than 90% of flagellum length (Figure 5) either in old flagella or in new flagella of cells treated for 20 h with teniposide. We propose that the available amount of pDC components is only 75% of what would be required to occupy the full length of the axoneme, explaining the published results (Edwards *et al*., 2018). However, in *lrrc56*^−^*^/^*^−^ cells, a low amount of dDC proteins are still present in the middle portion of the axoneme, spreading over ∼50% of the length but with a 2- to 3-fold lower density compared with controls (Figure 3C), suggesting that ∼20–25% of the binding sites on axonemal microtubules are occupied by the dDC. In these conditions, the amount of pDC would be sufficient to diffuse slowly and to progressively fill in the rest of the axoneme, explaining the >90% occupancy observed in old flagella or in new flagella of cells treated with teniposide.

In *C. reinhardtii*, the transport of ODAs could be visualized directly following the tagging of the IC2/DNAI2 component with the fluorescent mNG protein (Dai *et al*., 2018). Unfortunately, we could not detect the transport either of GFP::DNAI1 or mNG::ODAB (a dynein heavy chain) in growing or mature flagella (Vincensini *et al*., 2018), hence restricting further evaluation of the direct role of LRRC56 in ODA transport. In *Chlamydomonas*, ODAs extracted from wild-type axonemes can stably bind in vitro to the axoneme from the *oda8* mutant that locks dynein arms all along the axoneme (Desai *et al*., 2015). By contrast, dynein arms purified from the *oda8* mutant could not attach to wild-type purified axoneme supporting a role for LRRC56 in the maturation of ODAs prior to or during association to the axoneme rather than an involvement in docking complex presence on microtubules (Desai *et al*., 2015).

The unexpected link between LRRC56 and the docking complex that we have highlighted here could be the explanation of the variable impact of LRRC56 mutation or deletion between humans, algae, and trypanosomes. Indeed, the composition of the docking complex has evolved differently in these three organisms. The DC was first discovered in *Chlamydomonas* where it is made of at least three proteins called ODA3, ODA1 (two proteins rich in coiled coils), and ODA14 (a calcium-binding protein). While the ODA14 protein seems evolutionary restricted to a few groups (Casey *et al*., 2003), the two other ones are reasonably well conserved in eukaryotes with motile cilia, although they sometimes show extensive divergence (Jerber *et al*., 2014). ODA3 is orthologous to the human CCDC151 and to the trypanosome dDC1 and pDC1 while ODA1 finds orthologous proteins in the human CCDC114 and the trypanosome dDC2 and pDC2. In human cells, two other proteins called ARMC4 and TTC25 are involved in the docking complex and are required for proper outer dynein arm trafficking and docking (Hjeij *et al*., 2014; Wallmeier *et al*., 2016). These have not been found in algae or trypanosomes. Therefore, this significant divergence in the composition of the docking complex could explain the adaptation of its interactions with LRRC56. The trypanosome situation is perhaps the most revealing one, with the dDC requiring LRRC56 for efficient presence in the flagellum while the pDC seems independent of LRRC56. By contrast, the composition of the docking complex appears homogeneous from base to tip in the flagellum of *Chlamydomonas* (Owa *et al*., 2014) and in human airway cilia (Hjeij *et al*., 2014).

No visible impact on dynein arm presence could be detected in the human cilia of a PCD patient with a splicing mutation on the *lrrc56* gene, predicted to encode a nonfunctional protein (Bonnefoy *et al*., 2018). Recently, another mutation was found in LRRC56, but the cilia of the patient have not been examined (Alasmari *et al*., 2022). If dynein arms are apparently intact, how to explain the altered ciliary beating pattern in this patient? Possibly LRRC56 contributes to more discrete functions for the interactions between dynein arms and the docking complex. For example, LRRC56 might be involved in the proper folding of the dynein arm but not in its attachment. In these conditions, more advanced imaging approaches, such as cryo-electron microscopy could be necessary to evaluate discrete defects, as performed recently to demonstrate the role of tubulin glycylation in the beating of the sperm flagellum (Gadadhar *et al*., 2021).

In conclusion, our results support the hypothesis that the *T. brucei* LRRC56 is a transient IFT cargo protein involved in the binding of the docking complex proteins dDC1 and dDC2 to the distal portion of the axoneme during construction of the new flagellum. The finding of this interaction with the docking complex was unexpected knowing that flagella of the *Chlamydomonas oda8* mutant can bind dynein arms extracted from wild-type cells, suggesting that the docking complex is present and functional in the absence of LRRC56 in this organism. The exhaustive divergence in the composition of the docking complex probably explains the different contributions of LRRC56 to the proper attachment of dynein arms to the docking complex between human, trypanosome, and alga axonemes.

## MATERIAL AND METHODS

Request a protocol through *Bio-protocol*.

### Trypanosome cell culture

Cells used for this work were derivatives of *T. brucei* strain Lister 427 and were cultured in SDM79 medium supplemented with hemin and 10% FCS (Brun and Schönenberger, 1979).

### Expression of endogenous tagged *T. brucei* proteins

N-terminus tagging of LRRC56 with mNG was carried out as described (Dean *et al*., 2015) using p2675-derived template plasmid (Kelly *et al*., 2007) to amplify the puromycin drug resistance cassette as well as the gene encoding the mNG fluorescent reporter using forward primers containing 80 nucleotides of the 5′ UTR (untranslated region) of *LRRC56* (Tb927.10.15260) followed by the 20 nucleotides plasmid primer binding sequence and reverse primers consisting of the first 80 nucleotides of the target *lrrc56* open reading frame (Dean *et al*., 2015) in reverse complement orientation, followed by the last 20 reverse complement nucleotides of the mNG gene without the stop codon. Endogenous N-terminus tagging of *IFT81* (a member of the IFT-B complex) (Bhogaraju *et al*., 2013; Kubo *et al*., 2016) with tandem Tomato (tdT) fluorescent reporter (Shaner *et al*., 2004) was carried out using p2845-derived plasmid to amplify the blasticidin drug resistance cassette as well as the tDT gene using forward primers containing 80 nucleotides of the 5′ UTR of IFT81 (Tb927.10.2640) followed by the 20 nucleotides plasmid primer binding sequence and reverse primers consisting of the first 80 nucleotides of the target *IFT81* open reading frame (Dean *et al*., 2015) in reverse complement orientation, followed by the last 20 reverse complement nucleotides of the *tdT* gene without the stop codon.

For the generation of the mNG::dDC1 and mNG::dDC2 expressing cell lines, the first 750 nucleotides of dDC1 (Tb927.5.1900) and dDC2 (Gene DB number Tb927.11.16090) without the ATG were chemically synthesized (GeneCust) and cloned in frame with the mNG gene (Shaner *et al*., 2013) within the HindIII and ApaI sites of p2675 vector (Kelly *et al*., 2007) to generate p2675mNGdDC1 and p2675mNGdDC2. Before nucleofection, plasmid constructs were linearized within the dDC1 and dDC2 sequence with the enzyme ClaI and XcmI, respectively. For the generation of the mNG::pDC1 cell lines, the p2675mNGdDC2 plasmid was used as a template to amplify the puromycin drug resistance cassette as well as the gene encoding the mNG fluorescent reporter and using forward primers containing 80 nucleotides of the 5′ UTR of pDC1 (Tb927.8.4400) followed by the 20 nucleotide plasmid primer binding sequence and reverse primers consisting of the first 80 nucleotides of the target pDC1 open reading frame (Dean *et al*., 2015) in reverse complement orientation, followed by the last 20 reverse complement nucleotides of the mNG gene without the stop codon.

Sterile DNA was obtained following ethanol precipitation, and quantification was performed with a nanodrop. Transfection in the appropriate cell lines was achieved by nucleofection using program X-014 of the AMAXA Nucleofector apparatus (Lonza) as described previously (Burkard *et al*., 2007) with 10 µg linearized plasmids or 5 µg PCR product for homologous recombination with the target allele (Dean *et al*., 2015). Transgenic cell lines were obtained following appropriate drug selection according to the recipient cells used, then cloned if required by serial dilution.

### Generation of cell lines for RNAi knockdown

The 2913 cell line expressing the T7 RNA polymerase and the tetracycline repressor has been described previously (Wirtz *et al*., 1999). For generation of the *dDC2^RNAi^* and *dDC1^RNAi^* cell lines, a 498 bp fragment of *dDC2* (Tb927.11.16090) and a 505 bp fragment of *dDC1 (*Tb927.5.1900), both flanked by HindIII (upstream) and XhoI (downstream) sites, were selected using the RNAit algorithm (http://dag.compbio.dundee.ac.uk/RNAit/), to ensure that the fragment lacked significant identity to other genes and to avoid cross-RNAi (Redmond *et al*., 2003). These fragments were generated by chemical synthesis by GeneCust Europe (Boynes, France) and cloned into the corresponding HindIII-XhoI site of the digested pZJM vector (Wang *et al*., 2000) allowing for tetracycline-inducible expression of dsRNA (double stranded RNA) generating RNAi upon transfection in the 2913 recipient cell line. The dsRNA is expressed from two tetracycline-inducible T7 promoters facing each other in the pZJM vector.

### Intraflagellar trafficking analysis

Double-tagged mNG::LRRC56 and tdT::IFT81 cells were used to image two-color fluorescence simultaneously in live cells. Live cells were spread on a glass slide, covered with a coverslip and immediately observed with a spinning-disk confocal microscope (UltraVIEW VoX; Perkin Elmer) equipped with an oil-immersion objective Plan Apochromat 100 × /1.57 NA (ZEISS). Videos were acquired using Volocity software with two EM-CCD cameras (ImagEM X2 C9100-23B, Hamamatsu) in streaming mode. The samples were kept at 27°C using a temperature-controlled chamber. Sequences of 15 s were acquired with an exposure time of 100 ms per frame in dual-color imaging mode. Kymographs were extracted and analyzed with Icy software (de Chaumont *et al*., 2012) using the plugin Kymograph Tracker v2.1.3, where the anterograde kymographs were separated from the whole kymographs to improve signal-to-noise ratio and analyzed as described (Chenouard *et al*., 2010; de Chaumont *et al*., 2012; Buisson *et al*., 2013). For kymograph extraction, the tdT::IFT81 signal was used as a reference to determine the region of interest. That same region of interest was applied to the mNG::LRCC56 signal of the corresponding cell.

After kymograph extraction, the Icy software was used for the image background subtraction of green and red anterograde kymographs, followed by the generation of the Manders’ coefficients between the two signals with the Colocalization Studio plugin, using the same regions of interest as for the speed and frequency measurements.

### Indirect IFA

Cultured trypanosomes were washed twice in SDM79 medium without serum and spread directly on poly-L-lysine–coated slides (Thermo Fisher Scientific, Menzel-Gläser), air dried, then fixed in methanol at −20°C for 5 min followed by a rehydration step in PBS for 15 min. For cytoskeleton analysis, cells were settled onto poly-L-lysine–coated slides, and cytoskeletons were prepared as described (Bonhivers *et al*., 2008) by extraction with 0.4% NP-40 in 100 mM Pipes, pH 6.9, 2 mM EGTA (ethylene glycol tetraacetic acid), 1 mM MgSO_4_, and 0.1 mM EDTA. Following two washes in PIPES, cells were fixed in methanol at −20°C for 5 min and then rehydrated for 10 min in PBS. For immunodetection, slides were incubated for 1 h at 37°C with the appropriate dilution of the first antibody in 0.1% BSA in PBS: mAb25 against the axonemal protein TbSAXO1 (Dacheux *et al*., 2012) a mouse polyclonal antiserum against DNAI1 (Duquesnoy *et al*., 2009), or the 32F6 IgG2c mAb against mNG protein (Chromotek). After several 5 min washes in PBS, species and subclass-specific secondary antibodies coupled to the appropriate fluorochrome (FITC, Alexa 488, Cy3 or Cy5, Jackson ImmunoResearch) were diluted 1/400 in PBS containing 0.1% BSA and were applied for 1 h at 37°C. After washing in PBS as indicated above, cells were stained with a 1 µg/ml solution of the DNA-dye DAPI (Roche) and mounted with Slowfade antifade reagent (Invitrogen). Slides were either stored at −20°C or immediately observed with a DMI4000 microscope (Leica) with a 100X objective (NA 1.4) using Hamamatsu ORCA-03G or Prime95B Photometrics cameras with an EL6000 (Leica) as the light source. Image acquisition was performed using Micro-manager software and images were analyzed using ImageJ (National Institutes of Health, Bethesda, MD).

### Teniposide treatment

For inhibition of cell division, the topoisomerase II inhibitor teniposide (Sigma SML0609), was dissolved in DMSO and added to trypanosome cultures at a final concentration of 200 μM (Robinson and Gull, 1991; Bertiaux *et al*., 2018b) during 20 h. As a control, an identical volume of DMSO was added to the flasks.

### Statistical analysis

GraphPad Prism 8 application was used for all analyses. All data were analyzed using an unpaired *t* test with Welch's correction, except for the quantification of the anterograde speed, which was analyzed using the Brown–Forsythe and Welch ANOVA test.

## Supplementary Material



## Abbreviations used:

dDC: distal docking complex
pDC: proximal docking complex
IFT: intraflagellar transport
LRRC56: leucine-rich repeat protein 56
ODA: outer dynein arm.

## References

[B1] Ahmed NT, Gao C, Lucker BF, Cole DG, Mitchell DR (2008). ODA16 aids axonemal outer row dynein assembly through an interaction with the intraflagellar transport machinery. *J Cell Biol* *183*, 313–322. 18852297 10.1083/jcb.200802025 PMC2568026

[B2] Alasmari BG, Saeed M, Alomari MA, Alsumaili M, Tahir AM (2022). Primary ciliary dyskinesia: Phenotype resulting from a novel variant of LRRC56 gene. *Cureus* *14*, e28472. 36176820 10.7759/cureus.28472 PMC9512311

[B3] Atkins M, Týč J, Shafiq S, Ahmed M, Bertiaux E, De Castro Neto AL, Sunter J, Bastin P, Dean SD, Vaughan S (2021). CEP164C regulates flagellum length in stable flagella. *J Cell Biol* *220* e202001160. 33165561 10.1083/jcb.202001160 PMC7833213

[B4] Berriman M, Ghedin E, Hertz-Fowler C, Blandin G, Renauld H, Bartholomeu DC, Lennard NJ, Caler E, Hamlin NE, Haas B, *et al*. (2005). The genome of the African trypanosome Trypanosoma brucei. *Science* *309*, 416–422. 16020726 10.1126/science.1112642

[B5] Bertiaux E, Mallet A, Fort C, Blisnick T, Bonnefoy S, Jung J, Lemos M, Marco S, Vaughan S, Trépout S, *et al*. (2018a). Bidirectional intraflagellar transport is restricted to two sets of microtubule doublets in the trypanosome flagellum. *J Cell Biol* *217*, 4284–4297. 30275108 10.1083/jcb.201805030 PMC6279389

[B6] Bertiaux E, Morga B, Blisnick T, Rotureau B, Bastin P (2018b). A grow-and-lock model for the control of flagellum length in Trypanosomes. *Curr Biol* *28*, 3802–3814.e3. 30449671 10.1016/j.cub.2018.10.031

[B7] Bhogaraju S, Cajanek L, Fort C, Blisnick T, Weber K, Taschner M, Mizuno N, Lamla S, Bastin P, Nigg EA, Lorentzen E (2013). Molecular basis of tubulin transport within the cilium by IFT74 and IFT81. *Science* *341*, 1009–1012. 23990561 10.1126/science.1240985 PMC4359902

[B8] Billington K, Halliday C, Madden R, Dyer P, Barker AR, Moreira-Leite FF, Carrington M, Vaughan S, Hertz-Fowler C, Dean S, *et al*. (2023). Genome-wide subcellular protein map for the flagellate parasite Trypanosoma brucei. *Nat Microbiol* *8*, 533–547. 36804636 10.1038/s41564-022-01295-6 PMC9981465

[B9] Bonhivers M, Nowacki S, Landrein N, Robinson DR. (2008). Biogenesis of the trypanosome endo-exocytotic organelle is cytoskeleton mediated. *PLoS Biol* *6*, e105. 18462016 10.1371/journal.pbio.0060105 PMC2365980

[B10] Bonnefoy S, Watson CM, Kernohan KD, Lemos M, Hutchinson S, Poulter JA, Crinnion LA, Berry I, Simmonds J, Vasudevan P, *et al*. (2018). Biallelic mutations in LRRC56, encoding a protein associated with intraflagellar transport, cause mucociliary clearance and laterality defects. *Am J Hum Genet* *103*, 727–739. 30388400 10.1016/j.ajhg.2018.10.003 PMC6218757

[B11] Branche C, Kohl L, Toutirais G, Buisson J, Cosson J, Bastin P (2006). Conserved and specific functions of axoneme components in trypanosome motility. *J Cell Sci* *119*, 3443–3455. 16882690 10.1242/jcs.03078

[B12] Broadhead R, Dawe HR, Farr H, Griffiths S, Hart SR, Portman N, Shaw MK, Ginger ML, Gaskell SJ, McKean PG, Gull K (2006). Flagellar motility is required for the viability of the bloodstream trypanosome. *Nature* *440*, 224–227. 16525475 10.1038/nature04541

[B13] Brun R, Schönenberger (1979). Cultivation and *in vitro* cloning or procyclic culture forms of Trypanosoma brucei in a semi-defined medium. Short communication. *Acta Trop* *36*, 289–292. 43092

[B14] Buisson J, Chenouard N, Lagache T, Blisnick T, Olivo-Marin JC, Bastin P (2013). Intraflagellar transport proteins cycle between the flagellum and its base. *J Cell Sci* *126*, 327–338. 22992454 10.1242/jcs.117069

[B15] Burkard G, Fragoso CM, Roditi I (2007). Highly efficient stable transformation of bloodstream forms of Trypanosoma brucei. *Mol Biochem Parasitol* *153*, 220–223. 17408766 10.1016/j.molbiopara.2007.02.008

[B16] Casey DM, Inaba K, Pazour GJ, Takada S, Wakabayashi K, Wilkerson CG, Kamiya R, Witman GB (2003). DC3, the 21-kDa subunit of the outer dynein arm-docking complex (ODA-DC), is a novel EF-hand protein important for assembly of both the outer arm and the ODA-DC. *Mol Biol Cell* *14*, 3650–3663. 12972554 10.1091/mbc.E03-01-0057 PMC196557

[B17] Chenouard N, Buisson J, Bloch I, Bastin P, Olivo-Marin JC (2010). Curvelet analysis of kymograph for tracking bi-directional particles in flurescence microscopy images. *2010 IEEE International Conference on Image Processing*. Hong Kong, China: IEEE.

[B18] Craft JM, Harris JA, Hyman S, Kner P, Lechtreck KF (2015). Tubulin transport by IFT is upregulated during ciliary growth by a cilium-autonomous mechanism. *J Cell Biol* *208*, 223–237. 25583998 10.1083/jcb.201409036 PMC4298693

[B19] Dacheux D, Landrein N, Thonnus M, Gilbert G, Sahin A, Wodrich H, Robinson DR, Bonhivers M (2012). A MAP6-related protein is present in protozoa and is involved in flagellum motility. *PLoS One* *7*, e31344. 22355359 10.1371/journal.pone.0031344 PMC3280300

[B20] Dai J, Barbieri F, Mitchell DR, Lechtreck KF (2018). In vivo analysis of outer arm dynein transport reveals cargo-specific intraflagellar transport properties. *Mol Biol Cell* *29*, 2553–2565. 30133350 10.1091/mbc.E18-05-0291 PMC6254574

[B21] de Chaumont F, Dallongeville S, Chenouard N, Hervé N, Pop S, Provoost T, Meas-Yedid V, Pankajakshan P, Lecomte T, Le Montagner Y, *et al*. (2012). Icy: an open bioimage informatics platform for extended reproducible research. *Nat Methods* *9*, 690–696. 22743774 10.1038/nmeth.2075

[B22] Dean S, Sunter J, Wheeler RJ, Hodkinson I, Gluenz E, Gull K (2015). A toolkit enabling efficient, scalable and reproducible gene tagging in trypanosomatids. *Open Biol* *5*, 140197. 25567099 10.1098/rsob.140197 PMC4313374

[B23] Desai PB, Freshour JR, Mitchell DR (2015). Chlamydomonas axonemal dynein assembly locus ODA8 encodes a conserved flagellar protein needed for cytoplasmic maturation of outer dynein arm complexes. *Cytoskeleton* *72*, 16–28. 25558044 10.1002/cm.21206 PMC4361367

[B24] Duquesnoy P, Escudier E, Vincensini L, Freshour J, Bridoux AM, Coste A, Deschildre A, de Blic J, Legendre M, Montantin G, *et al*. (2009). Loss-of-function mutations in the human ortholog of Chlamydomonas reinhardtii ODA7 disrupt dynein arm assembly and cause primary ciliary dyskinesia. *Am J Hum Genet* *85*, 890–896. 19944405 10.1016/j.ajhg.2009.11.008 PMC2790569

[B25] Edwards BFL, Wheeler RJ, Barker AR, Moreira-Leite FF, Gull K, Sunter JD (2018). Direction of flagellum beat propagation is controlled by proximal/distal outer dynein arm asymmetry. *Proc Natl Acad Sci USA* *115*, E7341–E7350. 30030284 10.1073/pnas.1805827115 PMC6077732

[B26] Farr H, Gull K (2012). Cytokinesis in trypanosomes. *Cytoskeleton* *69*, 931–941. 23027706 10.1002/cm.21074

[B27] Fort C, Bonnefoy S, Kohl L, Bastin P (2016). Intraflagellar transport is required for the maintenance of the trypanosome flagellum composition but not its length. *J Cell Science* *129*, 3026–3041. 27343245 10.1242/jcs.188227

[B28] Gadadhar S, Alvarez Viar G, Hansen JN, Gong A, Kostarev A, Ialy-Radio C, Leboucher S, Whitfield M, Ziyyat A, Touré A, *et al*. (2021). Tubulin glycylation controls axonemal dynein activity, flagellar beat, and male fertility. *Science* *371*, eabd4914. 33414192 10.1126/science.abd4914 PMC7612590

[B29] Hjeij R, Onoufriadis A, Watson CM, Slagle CE, Klena NT, Dougherty GW, Kurkowiak M, Loges NT, Diggle CP, Morante NF, *et al*. (2014). CCDC151 mutations cause primary ciliary dyskinesia by disruption of the outer dynein arm docking complex formation. *Am J Hum Genet* *95*, 257–274. 25192045 10.1016/j.ajhg.2014.08.005 PMC4157146

[B30] Huet D, Blisnick T, Perrot S, Bastin P (2014). The GTPase IFT27 is involved in both anterograde and retrograde intraflagellar transport. *Elife* *3*, e02419. 24843028 10.7554/eLife.02419 PMC4003483

[B31] Jerber J, Baas D, Soulavie F, Chhin B, Cortier E, Vesque C, Thomas J, Durand B (2014). The coiled-coil domain containing protein CCDC151 is required for the function of IFT-dependent motile cilia in animals. *Hum Mol Genet* *23*, 563–577. 24067530 10.1093/hmg/ddt445

[B32] Kamiya R (1988). Mutations at twelve independent loci result in absence of outer dynein arms in Chylamydomonas reinhardtii. *J Cell Biol* *107*, 2253–2258. 2974040 10.1083/jcb.107.6.2253 PMC2115671

[B33] Kelly S, Reed J, Kramer S, Ellis L, Webb H, Sunter J, Salje J, Marinsek N, Gull K, Wickstead B, Carrington M (2007). Functional genomics in Trypanosoma brucei: a collection of vectors for the expression of tagged proteins from endogenous and ectopic gene loci. *Mol Biochem Parasitol* *154*, 103–109. 17512617 10.1016/j.molbiopara.2007.03.012 PMC2705915

[B34] King SM (2016). Axonemal dynein arms. *Cold Spring Harb Perspect Biol* *8*, a028100. 27527589 10.1101/cshperspect.a028100 PMC5088525

[B35] Knowles MR, Leigh MW, Ostrowski LE, Huang L, Carson JL, Hazucha MJ, Yin W, Berg JS, Davis SD, Dell SD, *et al*. (2013). Exome sequencing identifies mutations in CCDC114 as a cause of primary ciliary dyskinesia. *Am J Hum Genet* *92*, 99–106. 23261302 10.1016/j.ajhg.2012.11.003 PMC3542458

[B36] Koutoulis A, Pazour GJ, Wilkerson CG, Inaba K, Sheng H, Takada S, Witman GB (1997). The Chlamydomonas reinhardtii ODA3 gene encodes a protein of the outer dynein arm docking complex. *J Cell Biol* *137*, 1069–1080. 9166407 10.1083/jcb.137.5.1069 PMC2136212

[B37] Kubo T, Brown JM, Bellve K, Craige B, Craft JM, Fogarty K, Lechtreck KF, Witman GB (2016). Together, the IFT81 and IFT74 N-termini form the main module for intraflagellar transport of tubulin. *J Cell Sci* *129*, 2106–2119. 27068536 10.1242/jcs.187120 PMC5506485

[B38] Lechtreck K (2022). Cargo adapters expand the transport range of intraflagellar transport. *J Cell Science* *135*, jcs260408. 36533425 10.1242/jcs.260408 PMC9845741

[B39] Legendre M, Zaragosi LE, Mitchison HM (2021). Motile cilia and airway disease. *Semin Cell Dev Biol* *110*, 19–33. 33279404 10.1016/j.semcdb.2020.11.007

[B40] Omran H, Kobayashi D, Olbrich H, Tsukahara T, Loges NT, Hagiwara H, Zhang Q, Leblond G, O'Toole E, Hara C, *et al*. (2008). Ktu/PF13 is required for cytoplasmic pre-assembly of axonemal dyneins. *Nature* *456*, 611–616. 19052621 10.1038/nature07471 PMC3279746

[B41] Onoufriadis A, Paff T, Antony D, Shoemark A, Micha D, Kuyt B, Schmidts M, Petridi S, Dankert-Roelse JE, Haarman EG, *et al*. (2013). Splice-site mutations in the axonemal outer dynein arm docking complex gene CCDC114 cause primary ciliary dyskinesia. *Am J Hum Genet* *92*, 88–98. 23261303 10.1016/j.ajhg.2012.11.002 PMC3542455

[B42] Owa M, Furuta A, Usukura J, Arisaka F, King SM, Witman GB, Kamiya R, Wakabayashi K (2014). Cooperative binding of the outer arm-docking complex underlies the regular arrangement of outer arm dynein in the axoneme. *Proc Natl Acad Sci USA* *111*, 9461–9466. 24979786 10.1073/pnas.1403101111 PMC4084445

[B43] Pennarun G, Escudier E, Chapelin C, Bridoux AM, Cacheux V, Roger G, Clément A, Goossens M, Amselem S, Duriez B (1999). Loss-of-function mutations in a human gene related to Chlamydomonas reinhardtii dynein IC78 result in primary ciliary dyskinesia. *Am J Hum Genet* *65*, 1508–1519. 10577904 10.1086/302683 PMC1288361

[B44] Qiu T, Roy S (2022). Ciliary dynein arms: Cytoplasmic preassembly, intraflagellar transport, and axonemal docking. *J Cell Physiol* *237*, 2644–2653. 35128656 10.1002/jcp.30689

[B45] Redmond S, Vadivelu J, Field MC (2003). RNAit: an automated web-based tool for the selection of RNAi targets in Trypanosoma brucei. *Mol Biochem Parasitol* *128*, 115–118. 12706807 10.1016/s0166-6851(03)00045-8

[B46] Robinson D, Beattie P, Sherwin T, Gull K (1991). Microtubules, tubulin, and microtubule-associated proteins of trypanosomes. *Methods Enzymol* *196*, 285–299. 2034124 10.1016/0076-6879(91)96027-o

[B47] Robinson DR, Gull K (1991). Basal body movements as a mechanism for mitochondrial genome segregation in the trypanosome cell cycle. *Nature* *352*, 731–733. 1876188 10.1038/352731a0

[B48] Shaner NC, Campbell RE, Steinbach PA, Giepmans BN, Palmer AE, Tsien RY (2004). Improved monomeric red, orange and yellow fluorescent proteins derived from Discosoma sp. red fluorescent protein. *Nat Biotechnol* *22*, 1567–1572. 15558047 10.1038/nbt1037

[B49] Shaner NC, Lambert GG, Chammas A, Ni Y, Cranfill PJ, Baird MA, Sell BR, Allen JR, Day RN, Israelsson M, *et al*. (2013). A bright monomeric green fluorescent protein derived from Branchiostoma lanceolatum. *Nat Methods* *10*, 407–409. 23524392 10.1038/nmeth.2413 PMC3811051

[B50] Sherwin T, Gull K (1989). The cell division cycle of Trypanosoma brucei brucei: timing of event markers and cytoskeletal modulations. *Philos Trans R Soc Lond B Biol Sci* *323*, 573–588. 2568647 10.1098/rstb.1989.0037

[B51] Silflow CD, Lefebvre PA (2001). Assembly and motility of eukaryotic cilia and flagella. Lessons from Chlamydomonas reinhardtii. *Plant Physiol* *127*, 1500–1507. 11743094 PMC1540183

[B52] Subota I, Julkowska D, Vincensini L, Reeg N, Buisson J, Blisnick T, Huet D, Perrot S, Santi-Rocca J, Duchateau M, *et al*. (2014). Proteomic analysis of intact flagella of procyclic Trypanosoma brucei cells identifies novel flagellar proteins with unique sub-localization and dynamics. *Mol Cell Proteomics* *13*, 1769–1786. 24741115 10.1074/mcp.M113.033357 PMC4083114

[B53] Takada S, Wilkerson CG, Wakabayashi K, Kamiya R, Witman GB (2002). The outer dynein arm-docking complex: composition and characterization of a subunit (oda1) necessary for outer arm assembly. *Mol Biol Cell* *13*, 1015–1029. 11907279 10.1091/mbc.01-04-0201 PMC99616

[B54] Vincensini L, Blisnick T, Bertiaux E, Hutchinson S, Georgikou C, Ooi CP, Bastin P (2018). Flagellar incorporation of proteins follows at least two different routes in trypanosomes. *Biol Cell* *110*, 33–47. 29148062 10.1111/boc.201700052

[B55] Wallmeier J, Shiratori H, Dougherty GW, Edelbusch C, Hjeij R, Loges NT, Menchen T, Olbrich H, Pennekamp P, Raidt J, *et al*. (2016). TTC25 deficiency results in defects of the outer dynein arm docking machinery and primary ciliary dyskinesia with left-right body asymmetry randomization. *Am J Hum Genet* *99*, 460–469. 27486780 10.1016/j.ajhg.2016.06.014 PMC4974089

[B56] Wang Z, Morris JC, Drew ME, Englund PT (2000). Inhibition of Trypanosoma brucei gene expression by RNA interference using an integratable vector with opposing T7 promoters. *J Biol Chem* *275*, 40174–40179. 11013266 10.1074/jbc.M008405200

[B57] Wirtz E, Leal S, Ochatt C, Cross GA (1999). A tightly regulated inducible expression system for conditional gene knock-outs and dominant-negative genetics in Trypanosoma brucei. *Mol Biochem Parasitol* *99*, 89–101. 10215027 10.1016/s0166-6851(99)00002-x

[B58] Wren KN, Craft JM, Tritschler D, Schauer A, Patel DK, Smith EF, Porter ME, Kner P, Lechtreck KF (2013). A differential cargo-loading model of ciliary length regulation by IFT. *Curr Biol* *23*, 2463–2471. 24316207 10.1016/j.cub.2013.10.044 PMC3881561

[B59] Zur Lage P, Newton FG, Jarman AP (2019). Survey of the ciliary motility machinery of drosophila sperm and ciliated mechanosensory neurons reveals unexpected cell-type specific variations: A model for motile ciliopathies. *Front Genet* *10*, 24. 30774648 10.3389/fgene.2019.00024 PMC6367277

