# Supplementary material for: LRRC56 is an IFT cargo required for assembly of the distal dynein docking complex in Trypanosoma brucei
Source: Mol Biol Cell. 2024 Jul 11;35(8):ar106. doi: 10.1091/mbc.E23-11-0425 (PMC11321045; doi:10.1091/mbc.E23-11-0425)
Supplement: Supplementary file 3 [file mbc-35-ar106-s001.pdf]

Supplemental Materials

Molecular Biology of the Cell

Bonnefoy *et al*.

Figure S1. Kymograph analysis of a second cell expressing tdT::IFT81 and mNG::LRRC56. (A) A still image of the tdT::IFT81 signal, with the region of interest used to extract the kymographs in magenta (top) and the 15-second temporal projection of tdT::IFT81 (bottom). Scale bar: 3 µm. (B) The tdT::IFT81 whole kymograph (left) anterograde kymograph (right) of the cell in (A). Horizontal scale bar: 3 µm, vertical scale bar: 3 s. (C) A still image of the mNG::LRRC56 signal (top) of the same cell in (A), with the region of interest shown in green, and the 15-second temporal projection of mNG::LRCC56 (bottom). Scale bar: 3 µm. (D) The whole kymograph (left) and anterograde kymograph (right) of the cell in (C). Horizontal scale bar: 3 µm, vertical scale bar: 3 s. (E) Merge of IFT81 and LRRC56 anterograde kymographs shown in (B) and (D), respectively. (F) Schematic showing an event of LRCC56 being transported by an IFT81 particle (white line), an LRCC56 dissociation (dashed green line) from an IFT81 particle (magenta) and an event of LRRC56 being captured from the arrested material (dotted green line) by an IFT81 particle (magenta). (G) The anterograde particle containing IFT81 and LRRC56 signals (arrowheads) highlighted in (F) (white line) was observed in still images of the cell in (A) and (C) at the indicated time points. The full sequence is in the movie S2. Scale bar: 3 µm.

Figure S2. Efficiency of dDC2 knockdown and impact on dDC1. *dDC2^{RNAi}* cells expressing mNG::dDC2 (A) or mNG::dDC1 proteins (B) were observed by live epifluorescence imaging. In non-induced cells (day 0, left panels), both proteins are localised in the distal half portion of the axoneme, as expected. Following 4 days of *dDC2* RNAi induction (day 4, right panels), a nearly complete loss of dDC2 axonemal staining was achieved reflecting knockdown efficiency (A). In *dDC2^{RNAi}*-induced cells, mNG::dDC1 was barely associated with the axoneme and most of the fusion protein was found in cytoplasmic accumulations (B). The images are normalized using ImageJ according to minimum and maximum pixel values.

Figure S3. Flagellum length is shorter in *lrrc56$^{-/-}$* cells and not rescued upon inhibition of cell division. (A) The length of the old (OF) and of the new (NF) flagellum was measured from phase contrast images of detergent-treated wild-type (WT, black) and *lrrc56$^{-/-}$* (blue) cells, and is represented as violin plots. Both old and new flagella are significantly shorter in the *lrrc56$^{-/-}$* cell line. (B) The length of old and new flagella was measured using phase contrast images of the flagellum in wild-type and *lrrc56$^{-/-}$* cytoskeletons, in the absence or presence of teniposide (T). Violin plots representing the ratio between the length of the new

flagellum (NF) and that of the old flagellum (OF) are shown in percentage. While the new flagellum reaches the length of the old flagellum in teniposide-treated control cells (black), inhibition of cell division has little, if any, impact in *lrrc56*$^{-/-}$ cells (blue).

Figure S4 Inhibition of cell division restores most of the outer dynein arms despite the absence of LRRC56. IFA of cytoskeletons extracted from *lrrc56*$^{-/-}$ cells labelled for dDC2 (A), pDC1 cells (B) or DNAI1 (C) in control (left panels) or teniposide-treated conditions for 18 hours (right panels as indicated). Distal tips of flagella are shown by white (monoflagellated or mature flagellum) or blue asterisks (new flagellum). The tip of each signal is indicated with a white arrow. In *lrrc56*$^{-/-}$ cells treated with teniposide, the mNG::pDC1 and DNAI1 signals reach the distal tip of both the mature and the new flagellum (B-C). In the mature flagellum, the mNG::dDC2 signal also spreads toward the tip. However, in the new flagellum, nearly no redistribution of the mNG::dDC2 signal toward the tip is observed. Scale bars: 5 µm.

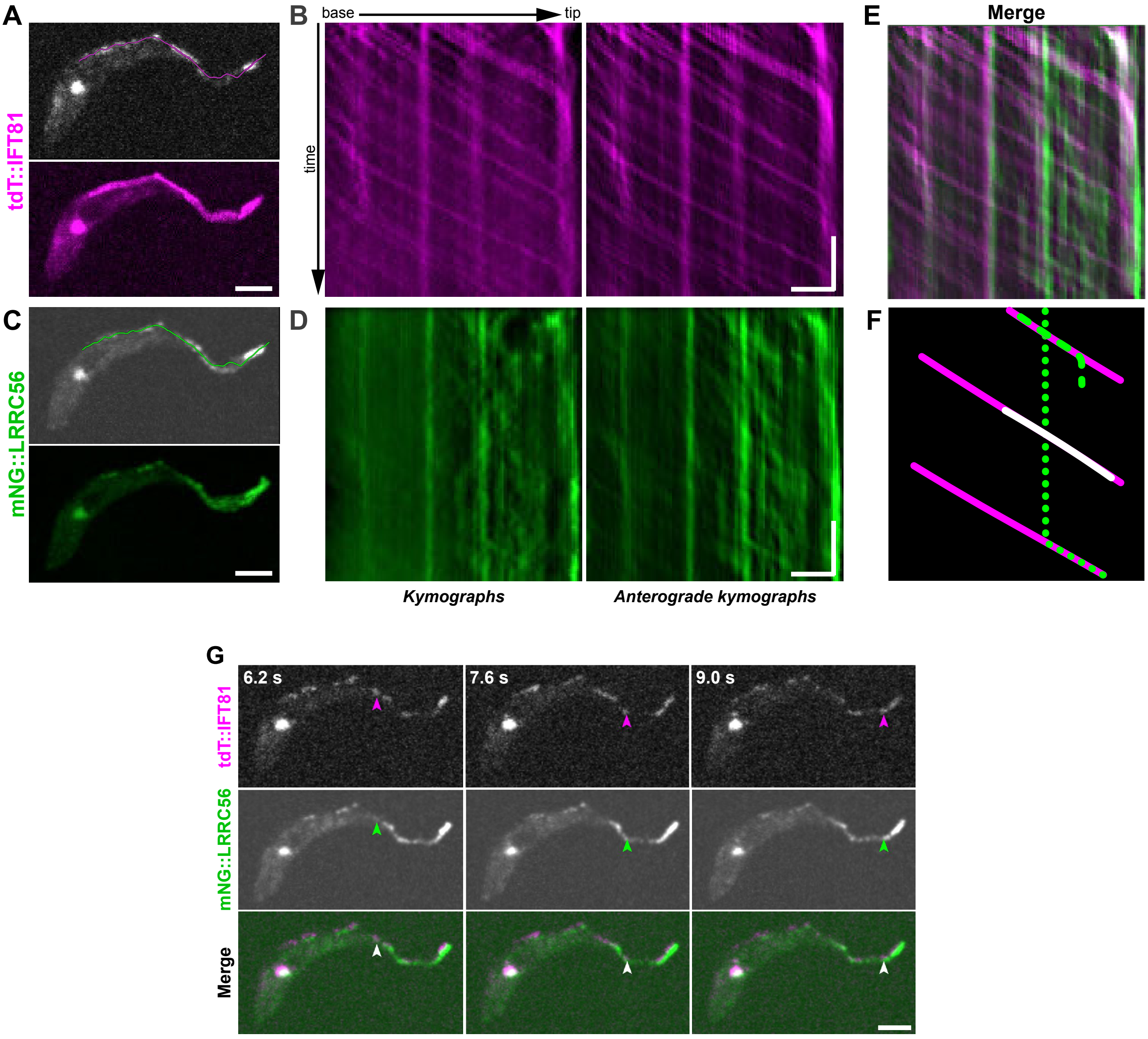
A
tdT::IFT81
B
base
tip
time
C
mNG::LRRC56
D
Kymographs
Anterograde kymographs
E
Merge
F
G
6.2 s
7.6 s
9.0 s
tdT::IFT81
mNG::LRRC56
Merge

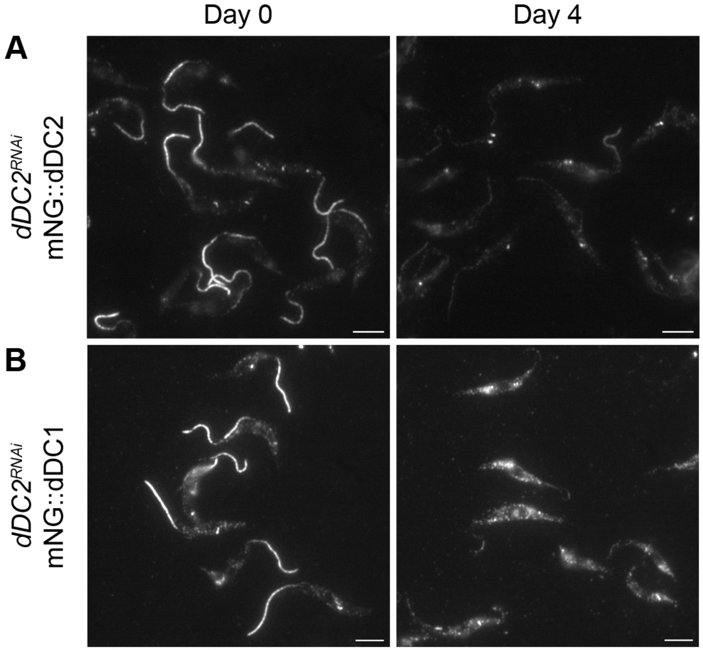

Day 0
Day 4
A
B
dDC2^RNAi mNG::dDC2
dDC2^RNAi mNG::dDC1

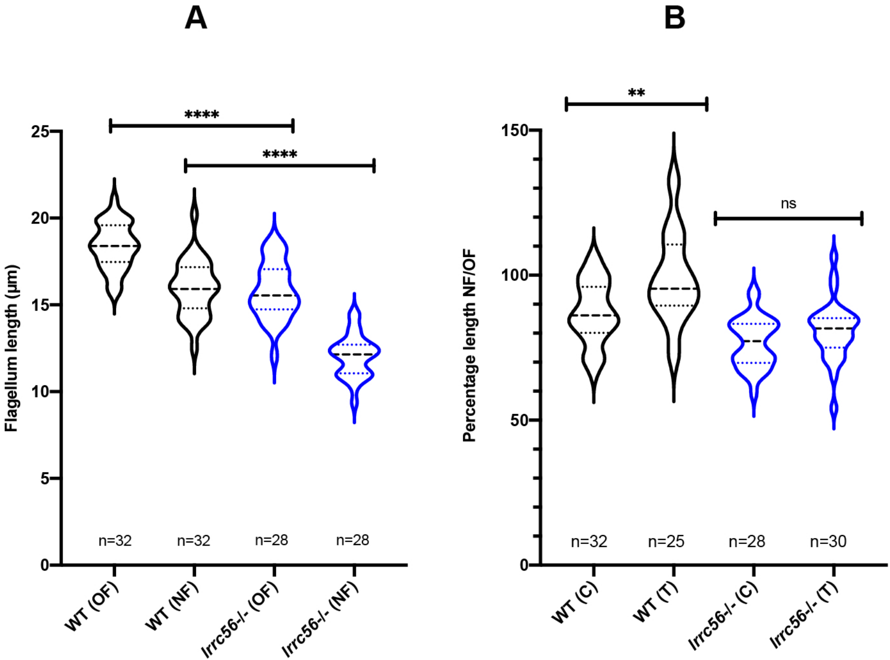
A
B
Flagellum length (µm)
25
20
15
10
5
0
****
****
n=32
n=32
n=28
n=28
WT (OF)
WT (NF)
lrrc56-/- (OF)
lrrc56-/- (NF)
Percentage length NF/OF
150
100
50
0
**
ns
n=32
n=25
n=28
n=30
WT (C)
WT (T)
lrrc56-/- (C)
lrrc56-/- (T)

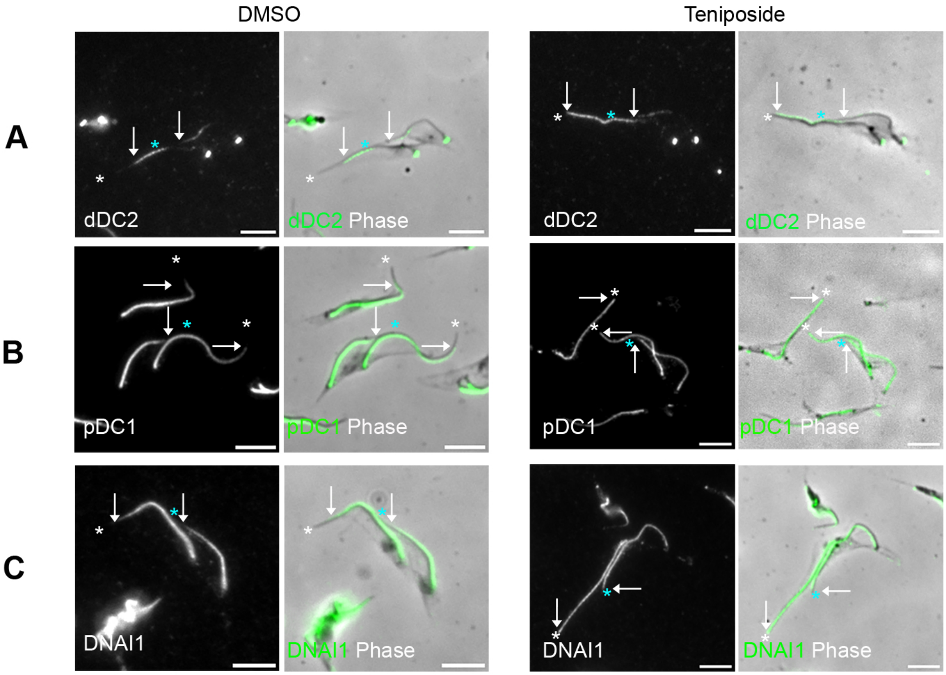
DMSO
Teniposide
A
B
C
dDC2
dDC2 Phase
pDC1
pDC1 Phase
DNAI1
DNAI1 Phase
