# Supplementary material for: LRRC56 is an IFT cargo required for assembly of the distal dynein docking complex in Trypanosoma brucei
Source: Mol Biol Cell. 2024 Jul 11;35(8):ar106. doi: 10.1091/mbc.E23-11-0425 (PMC11321045; doi:10.1091/mbc.E23-11-0425)
Supplement: Supplementary file 4 [file reviewer_reportE23-11-0425.pdf]

LRRC56 is an IFT cargo required for assembly of the distal dynein docking complex in Trypanosoma brucei

Serge Bonnefoy, Aline Alves, Eloise Bertiaux, and Philippe Bastin

Corresponding author(s): Serge Bonnefoy, Institut Pasteur

Review Timeline:

Submission Date:	2023-11-14
Editorial Decision:	2023-12-13
Revision Received:	2024-03-08
Editorial Decision:	2024-04-28
Revision Received:	2024-05-11
Editorial Decision:	2024-05-13
Revision Received:	2024-05-24
Accepted:	2024-05-25

Editor-in-Chief: Matthew Welch

Transaction Report:

(Note: With the exception of the correction of typographical or spelling errors that could be a source of ambiguity, letters and reports are not edited. The original formatting of letters and referee reports may not be reflected in this compilation.)

RE: Manuscript #E23-11-0425
TITLE: Novel roles for the LRRC56 protein, an IFT cargo protein, in docking of dynein arms in Trypanosoma brucei

Dear Dr. Bonnefoy,

Your manuscript has been reviewed by two experts in the field. Both reviewers thought that this work contain interesting new findings and that major conclusions are well supported. They also both made very constructive suggestions that will help to significantly improve data presentation, interpretation, and readability of the story. Please incorporate these suggestions in the revised manuscript as I think this will help to clarify the manuscript and figures. If you disagree with some of the suggestions, please explain the reason in the rebuttal letter.

The editors would also like to suggest revising the Significance Statement as follows:

In contrast to classic components of the flagellar outer dynein arms (ODAs), deletion or mutations of the LRRC56 protein in different species resulted in distinct phenotypes. [author - please state an unknown or open question]

A combination of functional genomics and live imaging reveals a molecular explanation to the distal absence of ODA in the protist Trypanosoma brucei and an unexpected interaction with components of the docking complex. Furthermore, LRRC56 is demonstrated to be a cargo of intraflagellar transport.

Because LRRC56 is mutated in some patients suffering from ciliopathies, the results are of significance for physiopathologists and cell biologists.

Best wishes,
Ke Hu
Monitoring Editor

Dear Dr. Bonnefoy,

The review of your manuscript, referenced above, is now complete. The Monitoring Editor has decided that your manuscript is not acceptable for publication at this time, but may be deemed acceptable after specific revisions are made, as described in the Monitoring Editor's decision letter above and the reviewer comments below.

A reminder: Please do not contact the Monitoring Editor directly regarding your manuscript. If you have any questions regarding the review process or the decision, please contact the MBoC Editorial Office (mboc@ascb.org).

When submitting your revision include a rebuttal letter that details, point-by-point, how the Monitoring Editor's and reviewers' comments have been addressed. (The file type for this letter must be "rebuttal letter"; do not include your response to the Monitoring Editor and reviewers in a "cover letter.") Please bear in mind that your rebuttal letter will be published with your paper if it is accepted, unless you have opted out of publishing the review history.

Authors are allowed 90 days to submit a revision. If this time period is inadequate, please contact us at mboc@ascb.org.

Revised manuscripts are assigned to the original Monitoring Editor whenever possible. However, special circumstances may preclude this. Also, revised manuscripts are often sent out for re-review, usually to the original reviewers when possible. The Monitoring Editor may solicit additional reviews if it is deemed necessary to render a completely informed decision.

In preparing your revised manuscript, please follow the instruction in the Information for Authors (www.molbiolcell.org/info-for-authors). In particular, to prepare for the possible acceptance of your revised manuscript, submit final, publication-quality figures with your revision as described.

To submit the rebuttal letter, revised manuscript, and figures, use this link: Link Not Available

MBoC offers the option to publish your work with immediate open access. Open access can increase the discoverability and usability of your research. If you would like to publish your paper with immediate open access but did not select this option during initial submission, please contact the editorial office at mbc@ascb.org.

Thank you for submitting your manuscript to MBoC. We look forward to receiving your revised paper.

Sincerely,

Eric Baker
Journal Production Manager
MBoC Editorial Office
mbc@ascb.org

Reviewer #1 (Remarks to the Author):

Report on Bonnefoy et al. "Novel roles for the LRRC56 protein ....".

This is a very nice follow-up study on the role of LRRC56 in the assembly of outer dynein arms (ODAs) and the dynein docking complex (DC) in Trypanosoma flagella. Work by the same first author previously established that pathogenic mutations in mammalian LRRC56 cause primary ciliary dyskinesia (PCD). Interestingly, loss of LRRC56 differentially affects the structure of the axoneme, i.e., the presence of outer dynein arms (ODAs), in different species. In human airway cilia from patients with mutations in LRRC56, ODAs are present but apparently not functional as indicated by the impairment of motility. In contrast, flagella of the Chlamydomonas oda8 mutants (ODA8 encodes Chlamydomonas LRRC56) largely lack ODAs. In the model studied here, biallelic loss of LRRC56 causes partial loss of ODAs, particularly from the distal region of flagella and from newly assembled flagella. Thus, further studies on LRRC56 have the potential to shed light on the conserved and specialized features of ODA assembly in different species with motile cilia.
Here, it is shown that LRRC56 moves by intraflagellar transport (IFT) without being a critical part of IFT. While IFT traffic is imaged routinely in various species, direct visualization of cargo transport represents a technical progress. Importantly, loss of LRRC56 interferes with the flagellar presence of the distal docking complex (dDC) proteins dDC1 and dDC2, which are reduced or lacking from the distal flagellar regions of the lrrc56-/- mutant flagella. The authors further demonstrate a mutual codependence as LRRC56 is reduced in flagella lacking the dDC. Loss of LRRC56 causes the proximal docking complex (pDC) to expand into the more distal territory, typically occupied by the dDC, as previously reported by others. Importantly, the authors demonstrate that the assembly of the pDC into more distal flagellar regions and the assembly of ODAs progresses with time. Indeed, ODA assembly can be largely restored when cell division is inhibited, which gives the new flagellum more time to mature. The data support the conclusions and are mostly clearly presented and discussed. Taken together, the work further characterizes an intriguing ciliary compensation mechanism that ensures the assembly of ODAs; it also highlights species-specific variations of LRRC56 function, ODA and DC assembly.
Below I have made some suggestions on how to improve the manuscript.

Main point
I think the description of mNG-LRRC56 trafficking by IFT needs to be improved. The authors state in the text "When observing the green channel, short fluorescent particles of mNG::LRRC56 displayed obvious anterograde movement (Figure 1Ac and Supplemental Movie S1). However, they appeared less processive than tdT::IFT81 and were mostly restricted to the distal portion of the flagellum (Figure 1Ac).". However, Fig. 1A d shows numerous mNG-LRRC56 tracks in the most proximal region of flagella.
The authors further state (e.g., in the figure legend) that "Continuous lines show IFT81 particles (magenta) carrying LRRC56 for a short part of their trajectory (white lines)." I am not sure that this interpretation is correct. The diagonal purple and white lines in panel f are supposed to indicate NG-LRRC56 transiently moving with dT-IFT81. To my eyes, it is pretty obvious that both proteins co-migrate not just in the white marked area but for most of the length of the cilia. It seems that the clearest of these co-transports (2nd from the top of the image) is not marked in panel f? The fact that the trajectories appear clearer while the trains move across a region with stationary NG-LRRC56 has probably technical reasons, i.e., strong combined signal or better focus. It is quite apparent, I think, that the LRRC56 trajectories are always clearer when the particles move across a field of stationary material. If unambiguous association or dissociation events between LRRC56 and IFT were observed, they should be shown and marked.
The paper also reports IFT-like movements of LRRC56 without co-migrating IFT81. It is mentioned that these particles move slower but the actual data are missing. Again, I wonder if these observations could have a technical, i.e., dT-IFT81 being bleached during illumination, rather than a biological basis. This idea is supported by the apparent lack of retrograde tracks for dT-IFT81. Previous studies from the same team documented retrograde IFT tracks in Trypanosoma. I recommend to reconsider the presentation of the data in the text and Figure 1 f.

Several figures (Figure 5 and subsequent figures) use ratios to describe the regions occupied by the pDC, dDC etc. However, it is unclear what, e.g., a ratio of 100 is supposed to mean. Ratios are typically expressed as 1:x and 1:100 would mean that one of the measurements is 100x larger. This is not the case here. May be using percent or just the real values in micro-meter is a better option.

Minor points
"However, the fact that ODA absence is less pronounced in mature flagella indicates that a timing issue could also be at stake in

the case of different growth rates for axoneme elongation and ODA addition in lrrc56-/- cells."
Phrase requires rewriting; "timing issue ...at stake" is unclear.

The authors document that the pDC expands progressively with time into the distal region of lrrc56-/- flagella likely explaining the presence of ODAs. However, Figure 3 B shows that the dDC is present in the distal flagellar regions of lrrc56-/- old flagella, suggesting that its assembly might be also time dependent. On page 21, it is mentioned that the inhibitor experiments were also carried out on strains expressing NG-dDC ("We have chemically blocked cell division in lrrc56-/- cells and control cells expressing mNG::dDC2, mNG::pDC1 or tdT::pDC1"). The corresponding data are shown only in Fig. 6B and original images are not shown. I wonder if the signal of the dDC increased in strength after teniposide treatment? Regardless of the outcome, the role of time for the LRRC56-independent assembly of the dDC should be briefly discussed.

Introduction P5: we considered two main hypotheses. First, the axoneme is heterogeneous all along its length. (...) Second, dynein arms are added to growing microtubules.
-> Heterogeneity along the length of flagella in Trypanosoma and the addition of ODAs to growing cilia are facts, not hypotheses. It appears that the two scenarios are not mutually exclusive. Needs, in my opinion, rewriting to more clearly state what the hypotheses are.

Discussion: "However, LRRC56 is able to move within the flagellum independently from IFT. and following sentences." As mention above, I think that technical issues, e.g., loss or failure to detect weaker IFT81 signals, could also explain these observations.

In Chlamydomonas, the absence of ODAs also causes a reduction of the docking complex in flagella. ("Overall, there was an 82% decrease (range 75-90%) in outer arm protein in mutant axonemes compared with wild type, but only a 25% decrease in the docking complex. The slight decrease in DC2 is consistent with the previous observation that oda6, an IC2 mutant that fails to assemble outer arm dyneins (Mitchell and Kang, 1991), also shows a slight reduction in docking complex proteins (Wakabayashi et al., 2001). The slight decrease in docking complex abundance, coupled with a much greater decrease in dynein motor subunits, is consistent with a role for ODA16p in assembly of the motor but not the docking complex."). Is it clear that the dDC is assembled independently of the ODAs in Trypanosoma? If not, this might be an additional thing to check.

Images: Please ensure that the stars and arrows do not cover up details of the image. In Fig. 3A, for example, the distal NG-dDC2 negative tip appears to be obscured. Cells in Fig. 2, bottom right appear better suited to show the absence of this marker from the very tips.

Fig. 5) What is meant by mNG::pdDC1? Should it be pDC1?

Title: Novel roles for the LRRC56 protein, an IFT cargo protein, in docking of dynein arms in Trypanosoma brucei
May be the role should be specified? May be "Trypanosoma LRRC56 is an IFT cargo required for flagellar assembly of the distal dynein docking complex?

Reviewer #2 (Remarks to the Author):

The authors have previously shown the T. brucei lrrc56 protein, is localized to the flagellum, where it overlaps with the IFT machinery, and that loss of lrrc56 in T brucei results in loss of dyneins in the axoneme. Similar results have also been reported in other organisms, though in T. brucei, the dynein defect was distinguished as being restricted to the distal ~1/2 of the axoneme. In the current work, they extend earlier studies, reporting active movement of lrrc56 with IFT particles in live trypanosomes. Additionally, they provide insight into the mechanisms of dynein disruption by reporting that lrrc56 is required for assembly of the distal docking complex (dDC1/2) at the extreme distal tip of the flagellum, indicating it is loss of dDC1/2 that is responsible for loss of dyneins and, further, that proximalDC (pDC) expands longer on the axoneme in lrrc56 mutants and may explain the retention of dynein in the trypanosome lrrc56 mutant vs other organisms. This is a very nice paper, reporting new mechanistic insights into flagellum assembly mechanisms in trypanosomes, and expanding understanding of the processes by which heterogeneity along the axoneme may be achieved, which is relevant beyond trypanosomes. I do have a some comments noted below that the authors should address in order to strengthen the work.

Concerns:
· It seems to me that there's a disconnect between loss of dDC and extension of pDC in the lrrc56 mutant. The authors suggest this is due to pDC now accessing unoccupied sites vacated by dDC (e.g. p19), but the data do not support this. In Fig 5B, for example lrrc56 loss causes slightly less dDC in OF, but enables alot more pDC. Therefore, a simple competition for binding sites, as suggested by the authors, cannot be the explanation for expansion of pDC to distal regions of the flagellum. Indeed, dDC remains in much of the distal flagellum with the exception of the extreme tip (Fig 3), while pDC (Fig 4) occupies much the same regions covered by dDC (Fig 3). Finally, if expansion of pDC is simply do to occupying open space vacated by dDC, there should be more pDC in NF than in OF, yet the opposite result is obtained.
· The "two models" put forth on top of p5 need to be better articulated. As written, these seem confusing or overly contrived. I find most of the discussion of "old" vs "new" flagellum results to be somewhat hard to follow and perhaps an oversimplification of

what is truly a dynamic process, e.g. extending an axoneme and adding many motors. I do not think the impact of this important paper would be diminished if the presentation of 'distinguishing between two models' were substantially curtailed or removed altogether.

· Several figures report "percentage of flagellum" covered by a labeled protein (e.g. Fig 5A, B). The relevant question is what portions along the flagellum are labeled, so a more informative representation of the data would be to indicate what portion, base to tip, of the flagellum is labeled. Note that representative images should also be shown in suppl data.

· All charts should show actual data points, e.g. as in Fig 1 D and E, rather than simple box and whiskers plots shown for most/all other charts. Even something like a violin plot would be more informative and realistic representation of the data.

· Authors mention shorter flagella in the abstract, but I didn't see any measurements of flagellum length (only the ratio of new/old).

· In Fig. 3 legend, they say 'A non-specific signal is also observed at the base of the flagella of tagged mNG::dDC2 cells, exactly as found on control cells (not shown)' but the control cells in Fig 2A don't seem to show this same non-specific signal.

· Text is confusing or odd in some places and should be gone through thoroughly to check for inconsistencies. For example:

○ Fig 1C. It is hard to reconcile the "significantly slower" speed claimed for lrrc56 vs IFT81 (p12) based on the plot shown in figure 1c, as the data indicate nearly identical speed, albeit broader distribution in speed for IFT81. Why does fig 1c shown as box and whiskers, instead of all the data points as shown for 1d and 1e? Should show all the data points?

○ Fig 2 and text on p15.
Absence of DC1/2 in the very distal tip of the flagellum in lrrc56 mutants is evident. But, the claim that singal intensity is "signfificantly reduced" is not evident. Either provide quantitation to support the claim, or remove.

○ Fig 3: arrows are white or yellw, but text says white or red.

○ Fig 3C and text on p16. Care should be taken in claiming the dDC2 in the flagellum 'remains' associated with the cytoskeleton. The statement implies all the dDC2 that gets there remains stably associated with the axoneme, but that's not what the data show. The data show dDC2 can be detected after detergent extraction, but it is not comparing before vs after extraction. Indeed, the signal in panel 3C looks much reduced compared to non-extracted cells shown in 3A.. Authors could assess this via western blot of detergent-solubliized vs detergent insoluble fractions. Otherwise, change the statement to indicate some dDC2 remains on the axoneme without implying it all does. Also: for fig 3D, please specify in figure legend whether intensity analysis was done on detergent-extracted samples.

○ Fig 5C/D is very confusing:
-labels at top indicate "pd" DC
-schematic indicates "p" DC
-figure legend says "expressing
mNG::pDC1 and mNG::pDC1", which is redundant.
What is being examined?

○ The suggestion on p3/4 that nuanced, species-specific differences in lrrc56 mutant defects is "unusual" seems a bit overstated. I think there are several examples of species-specific differences in phenotype for other flagellar proteins.

○ p4, near end of page: authors say LRRC56 is found in the flagellum MATRIX. Do they mean matrix, as in SOLUBLE, or do they mean to say axoneme? or is it both? Answering this would require authors to do comparison of LRRC56 distribution between supe and pellet from detergent-extracted cells. (this is relevant to comments elsewhere concerning how much of the lrrc56 protein remains associated with the axoneme upon detergent extraction)

○ Please explain the statement on p12 that LRRC56 is absent from mature flagella.

○ P12, statement that Fig 1A,B provide the "first" evidence for lrrc56 as IFT cargo. This doesn't seem to be correct, because these authors showed previously that lrr56 co-localized with IFT particles, and I think some data have shown LRRC56 to coIP with IFT proteins. So, the current data show movement, but it's not the first evidence...

○ In the 1st paragraph of results, authots say 'Acquisitions were performed on monoflagellated cells displaying an mNG::LRRC56 fluorescent signal, therefore corresponding to cells that inherited a new flagellum after cytokinesis, LRRC56 being absent from the mature flagellum.' I don't understand this comment - please clarify.

○ p. 24 'dramatic loss of mNG::dDC1 signal was observed': Are the authors saying that the cytoplasmic dDC1 signal is reduced in intensity compared to controls? It is not clear based on how they scaled their images whether overall signal is decreased or whether there is mislocalization to the cytoplasm. If they are referring to loss of flagellar signal, that should be clarified

○ Fig S1: I'm not familiar with how one does 'background subtraction' on kymographs to get just the anterograde ones. Can the authors elaborate in the methods?

Manuscript #E23-11-0425: point by point response letter

TITLE: Novel roles for the LRRC56 protein, an IFT cargo protein, in docking of dynein arms in *Trypanosoma brucei*

We thank the editor and the reviewers for their interest, support and constructive suggestions. Our responses are below in blue.

Response to editor suggestions

The editors would also like to suggest revising the Significance Statement as follows:

In contrast to classic components of the flagellar outer dynein arms (ODAs), deletion or mutations of the LRRC56 protein in different species resulted in distinct phenotypes.
[author - please state an unknown or open question]

We are proposing the following (page 3):

Why do mutations of the LRRC56 protein have distinct impacts on the organization of outer dynein arms (ODAs) between ciliated species?

- A combination of functional genomics and live imaging reveals a molecular explanation for the distal absence of ODA in the protist *Trypanosoma brucei* and an unexpected interaction with components of the docking complex.
- Furthermore, LRRC56 is demonstrated to be a cargo of intraflagellar transport.
- Because LRRC56 is mutated in some patients suffering from ciliopathies, the results are of significance not only for cell biologists but also for physio-pathologists

We thank both reviewers for their enthusiastic feedback, as well as their constructive and useful suggestions. We have addressed all their comments as described in the point-by-point response letter.

Reviewer #1 (Remarks to the Author):

Report on Bonnefoy et al. "Novel roles for the LRRC56 protein ....".

This is a very nice follow-up study on the role of LRRC56 in the assembly of outer dynein arms (ODAs) and the dynein docking complex (DC) in Trypanosoma flagella. Work by the same first author previously established that pathogenic mutations in mammalian LRRC56 cause primary ciliary dyskinesia (PCD). Interestingly, loss of LRRC56 differentially affects the structure of the axoneme, i.e., the presence of outer dynein arms (ODAs), in different species. In human airway cilia from patients with mutations in LRRC56, ODAs are present but apparently not functional as indicated by the impairment of motility. In contrast, flagella of the Chlamydomonas oda8 mutants (ODA8 encodes Chlamydomonas LRRC56) largely lack ODAs. In the model studied here, biallelic loss of LRRC56 causes partial loss of ODAs, particularly from the distal region of flagella and from newly assembled flagella. Thus, further studies on LRRC56 have the potential to shed light on the conserved and specialized features of ODA assembly in different species with motile cilia.

Here, it is shown that LRRC56 moves by intraflagellar transport (IFT) without being a critical part of IFT. While IFT traffic is imaged routinely in various species, direct visualization of cargo transport represents a technical progress. Importantly, loss of LRRC56 interferes with the flagellar presence of the distal docking complex (dDC) proteins dDC1 and dDC2, which are reduced or lacking from the distal flagellar regions of the lrrc56-/- mutant flagella. The authors further demonstrate a mutual codependence as LRRC56 is reduced in flagella lacking the dDC. Loss of LRRC56 causes the proximal docking complex (pDC) to expand into the more distal territory, typically occupied by the dDC, as previously reported by others. Importantly, the authors demonstrate that the assembly of the pDC into more distal flagellar regions and the assembly of ODAs progresses with time. Indeed, ODA assembly can be largely restored when cell division is inhibited, which gives the new flagellum more time to mature. The data support the conclusions and are mostly clearly presented and discussed. Taken together, the work further characterizes an intriguing ciliary compensation mechanism that ensures the assembly of ODAs; it also highlights species-specific variations of LRRC56 function, ODA and DC assembly.

Below I have made some suggestions on how to improve the manuscript.

Main point I think the description of mNG-LRRC56 trafficking by IFT needs to be improved.

We thank the reviewer for this detailed analysis. We agree with all the points raised below and have significantly revised the text and figures 1 and S1 for a more precise description.

The authors state in the text "When observing the green channel, short fluorescent particles of mNG::LRRC56 displayed obvious anterograde movement (Figure 1Ac and Supplemental Movie S1). However, they appeared less processive than tdT::IFT81 and were mostly restricted to the distal portion of the flagellum (Figure 1Ac).". However, Fig. 1A d shows numerous mNG-LRRC56 tracks in the most proximal region of flagella.

We have revisited our series of videos of mNG::LRRC56/tdT::IFT81 cells and confirmed that LRRC56 indeed traffics from the base to the tip of the flagellum. These events have been now highlighted in the revised Figure 1. The sentence has been rewritten (page 8) as “When observing the green channel, short fluorescent particles of mNG::LRRC56 displayed obvious anterograde movement (Figure 1Ac and Supplemental Movie S1), showing displacement

along the whole flagellum length, as expected for a conventional IFT cargo (continuous white line, Figure 1Af).

The authors further state (e.g., in the figure legend) that "Continuous lines show IFT81 particles (magenta) carrying LRRC56 for a short part of their trajectory (white lines)." I am not sure that this interpretation is correct. The diagonal purple and white lines in panel f are supposed to indicate NG-LRRC56 transiently moving with dT-IFT81. To my eyes, it is pretty obvious that both proteins co-migrate not just in the white marked area but for most of the length of the cilia. It seems that the clearest of these co-transports (2nd from the top of the image) is not marked in panel f? The fact that the trajectories appear clearer while the trains move across a region with stationary NG-LRRC56 has probably technical reasons, i.e., strong combined signal or better focus.

We considered this carefully but we could indeed find multiple traces of mNG::ODA8 without tdT::IFT81 (see figure 1Af, frequency analysis, Figure 1D and Manders analysis, Figure 1E). Technical limitations sound unlikely for several reasons: (1) the signal for mNG::ODA8 is much weaker than the one for tdT::IFT81, (2) although only one allele of *IFT81* is tagged (so in theory 50% of the total pool of IFT81), the presence of at least 30 copies per IFT train (Bertiaux JCB2018; Jordan NCB2018) means that all the IFT trains should be labelled and (3) since it is simultaneous acquisition with a dual beam splitter if a region of the flagellum is out of focus, this will be the case for both channels.

The clearest of the co-transport event mentioned by the reviewer is now highlighted as a white line in Figure 1Af and described on page 8 (see above).

It is quite apparent, I think, that the LRRC56 trajectories are always clearer when the particles move across a field of stationary material. If unambiguous association or dissociation events between LRRC56 and IFT were observed, they should be shown and marked.

We looked for these events and could indeed find both of them (LRRC56 being “dropped” from or “caught up” by IFT trains in about half of the videos. These events are now clearly marked in Figure S1f and discussed in the text (page 9) and figure legends.

The paper also reports IFT-like movements of LRRC56 without co-migrating IFT81. It is mentioned that these particles move slower but the actual data are missing.

The higher frequency of mNG::LRRC56 compared to tdT::IFT81 and the relatively low Manders coefficient supported this point but to show this more directly, the data is now quantified and displayed in Fig. 1C, where the speed of moving mNG::LRRC56 particles not associated with IFT trains (grey violin) is now shown separately from those associated to tdT::IFT81 (green violin). This is also mentioned in the text (pages 8-9).

Again, I wonder if these observations could have a technical, i.e., dT-IFT81 being bleached during illumination, rather than a biological basis. This idea is supported by the apparent lack of retrograde tracks for dT-IFT81. Previous studies from the same team documented retrograde IFT tracks in Trypanosoma. I recommend to reconsider the presentation of the data in the text and Figure 1 f.

As said above, it is unlikely that the IFT signal bleaches faster since it is much brighter than the mNG::LRRC56 signal. We focused here on anterograde transport (see Figure 1 and its

legend) since the retrograde traces are too weak to be analysed. We apologize if this was not clear and we are now stating this more clearly in the text (page 8 “Retrograde traces were sometimes detected but were too weak to be analysed with confidence, the analysis was therefore focused on anterograde movement.”) and in the M&M section (page 21 “the anterograde kymographs were separated from the whole kymographs and analyzed”). A full kymograph, presented side-by-side with the extracted anterograde kymograph, is shown in Figure S1b/d.

There are indeed no retrograde traces on the kymographs since only the anterograde signal was extracted to reduce a background, a standard procedure used in the field and described specifically in several publications (Chenouard et al. 2010, Buisson et al., JCS2013).

Several figures (Figure 5 and subsequent figures) use ratios to describe the regions occupied by the pDC, dDC etc. However, it is unclear what, e.g., a ratio of 100 is supposed to mean. Ratios are typically expressed as 1:x and 1:100 would mean that one of the measurements is 100x larger. This is not the case here. May be using percent or just the real values in micro-meter is a better option.

We have changed as suggested the Y-axis legend of all the figures using percentages instead of ratios. Data representation has been changed using violin plots in all figures as suggested by referee 2.

Minor points

"However, the fact that ODA absence is less pronounced in mature flagella indicates that a timing issue could also be at stake in the case of different growth rates for axoneme elongation and ODA addition in lrrc56-/- cells."
Phrase requires rewriting; "timing issue ...at stake" is unclear.

“This sentence has been modified as: “However, the fact that ODA absence is less pronounced in mature flagella indicates that axoneme elongation and ODA addition might not take place at the same rate in *lrrc56*$^{-/-}$ cells, with possibly further addition of ODA after cell division” (page 12).

The authors document that the pDC expands progressively with time into the distal region of lrrc56-/- flagella likely explaining the presence of ODAs. However, Figure 3 B shows that the dDC is present in the distal flagellar regions of lrrc56-/- old flagella, suggesting that its assembly might be also time dependent. On page 21, it is mentioned that the inhibitor experiments were also carried out on strains expressing NG-dDC ("We have chemically blocked cell division in lrrc56-/- cells and control cells expressing mNG::dDC2, mNG::pDC1 or tdT::pDC1"). The corresponding data are shown only in Fig. 6B and original images are not shown Again, I wonder if the signal of the dDC increased in strength after teniposide treatment?

A new figure (Fig. S4) has been added to show representative images of cells treated with teniposide with the localization of the distal DC (dDC2), the proximal DC (pDC1) and outer dynein arms (DNA1), in addition to the previous Figure 6C-D. Indeed, teniposide treatment induces a slight increase in the distribution of the dDC2 protein but only in old flagella (Figure S4A, sentence added page 13).

Regardless of the outcome, the role of time for the LRRC56-independent assembly of the dDC should be briefly discussed.

This is indeed correct and it is now discussed on page 13: “It should be noted that the distal docking complex also seems to expand further during flagellum maturation, as seen with a longer portion of brighter signal for mNG::dDC2 in old flagella compared to new flagella (Fig. 3B-D).”

Introduction P5: we considered two main hypotheses. First, the axoneme is heterogeneous all along its length. (...) Second, dynein arms are added to growing microtubules. -> Heterogeneity along the length of flagella in Trypanosoma and the addition of ODAs to growing cilia are facts, not hypotheses. It appears that the two scenarios are not mutually exclusive. Needs, in my opinion, rewriting to more clearly state what the hypotheses are.

We rewrote this section to present the hypotheses more concisely (also in response to reviewer 2): “we propose two hypotheses (not necessarily exclusive): (1) LRRC56 could only be required for the addition of dynein arms to the distal docking complex and (2) LRRC56 could be required for efficient entry, transport or maturation of dynein arms in the trypanosome flagellum as observed in *Chlamydomonas* (Dai *et al.*, 2018).”. We also rewrote the presentation of flagellum heterogeneity as facts as proposed by the reviewer: “we considered the peculiarities of flagellum composition and assembly in *T. brucei*. It is known that the axoneme is heterogeneous all along its length”. (top page 6)

Discussion: "However, LRRC56 is able to move within the flagellum independently from IFT. and following sentences." As mention above, I think that technical issues, e.g., loss or failure to detect weaker IFT81 signals, could also explain these observations.

As discussed above, this looks very unlikely since the IFT81 signal is much brighter than the mNG::LRRC56 signal and simultaneous acquisition is performed with a dual beam splitter.

In Chlamydomonas, the absence of ODAs also causes a reduction of the docking complex in flagella. ("Overall, there was an 82% decrease (range 75-90%) in outer arm protein in mutant axonemes compared with wild type, but only a 25% decrease in the docking complex. The slight decrease in DC2 is consistent with the previous observation that oda6, an IC2 mutant that fails to assemble outer arm dyneins (Mitchell and Kang, 1991), also shows a slight reduction in docking complex proteins (Wakabayashi et al., 2001). The slight decrease in docking complex abundance, coupled with a much greater decrease in dynein motor subunits, is consistent with a role for ODA16p in assembly of the motor but not the docking complex."). Is it clear that the dDC is assembled independently of the ODAs in Trypanosoma? If not, this might be an additional thing to check.

To our knowledge, the question of independent association of the docking complex from the ODAs in *T. brucei* has not been addressed. As pointed out by the reviewer, a significant amount of docking complex proteins is added to flagellar microtubules in the absence of ODA in *Chlamydomonas*, as supported by several published observations that some small protrusions, likely corresponding to the docking complex are present in ODA mutants. These protrusions as well as ODAs are absent in *oda1* and *oda3* docking complex mutants (Takada *et al*, JCB1994). We re-examined our electron microscopy images of axonemes from the *DNAI1*^{RNAi} cell line (outer dynein arm component whose ablation results in the loss of ODAs, (Branche *et al*, JCS 2006). They indeed display an absence of ODAs but small protrusions potentially corresponding to remnants of the DC could not be detected. Furthermore, the loading of dynein arms on IFT trains and incorporation into the axoneme are likely to operate differently in *T. brucei*. Knockdown of the ODA16 adapter protein does not show obvious ODA defects by transmission electron microscopy and little or no decrease of ODA components (Huang *et al*, BioRXiV2023).

Images: Please ensure that the stars and arrows do not cover up details of the image. In Fig. 3A, for example, the distal NG-dDC2 negative tip appears to be obscured. Cells in Fig. 2, bottom right appear better suited to show the absence of this marker from the very tips.

All figure annotations have been modified accordingly

Fig. 5) What is meant by mNG::pdDC1? Should it be pDC1?

This was a typing error and has been corrected.

Title: Novel roles for the LRRC56 protein, an IFT cargo protein, in docking of dynein arms in Trypanosoma brucei

May be the role should be specified? May be "Trypanosoma LRRC56 is an IFT cargo required for flagellar assembly of the distal dynein docking complex?

Thanks for the suggestions, we propose:

LRRC56 is an IFT cargo required for assembly of the distal dynein docking complex in *Trypanosoma brucei*

Reviewer #2 (Remarks to the Author):

The authors have previously shown the T. brucei lrrc56 protein, is localized to the flagellum, where it overlaps with the IFT machinery, and that loss of lrrc56 in T brucei results in loss of dyneins in the axoneme. Similar results have also been reported in other organisms, though in T. brucei, the dynein defect was distinguished as being restricted to the distal ~1/2 of the axoneme. In the current work, they extend earlier studies, reporting active movement of lrrc56 with IFT particles in live trypanosomes. Additionally, they provide insight into the mechanisms of dynein disruption by reporting that lrrc56 is required for assembly of the distal docking complex (dDC1/2) at the extreme distal tip of the flagellum, indicating it is loss of dDC1/2 that is responsible for loss of dyneins and, further, that proximalDC (pDC) expands longer on the axoneme in lrrc56 mutants and may explain the retention of dynein in the trypanosome lrrc56 mutant vs other organisms. This is a very nice paper, reporting new mechanistic insights into flagellum assembly mechanisms in trypanosomes, and expanding understanding of the processes by which heterogeneity along the axoneme may be achieved, which is relevant beyond trypanosomes. I do have a some comments noted below that the authors should address in order to strengthen the work.

We thank the reviewer for this supportive analysis and useful suggestions that are addressed below.

Concerns:
· It seems to me that there's a disconnect between loss of dDC and extension of pDC in the lrrc56 mutant. The authors suggest this is due to pDC now accessing unoccupied sites vacated by dDC (e.g. p19), but the data do not support this. In Fig 5B, for example lrrc56 loss causes slightly less dDC in OF, but enables alot more pDC. Therefore, a simple competition for binding sites, as suggested by the authors, cannot be the explanation for expansion of pDC to distal regions of the flagellum. Indeed, dDC remains in much of the distal flagellum

with the exception of the extreme tip (Fig 3), while pDC (Fig 4) occupies much the same regions covered by dDC (Fig 3). Finally, if expansion of pDC is simply do to occupying open space vacated by dDC, there should be more pDC in NF than in OF, yet the opposite result is obtained.

The explanation probably lies in the resolution of optical microscopy. The dDC components occupy a more restricted portion than in control cells as clearly shown in Figures 2 and 3. Moreover, even at positions occupied by dDC components, their fluorescence intensity is much reduced, suggesting that they are not present on all 9 doublets.

· The "two models" put forth on top of p5 need to be better articulated. As written, these seem confusing or overly contrived. I find most of the discussion of "old" vs "new" flagellum results to be somewhat hard to follow and perhaps an oversimplification of what is truly a dynamic process, e.g. extending an axoneme and adding many motors. I do not think the impact of this important paper would be diminished if the presentation of 'distinguishing between two models' were substantially curtailed or removed altogether.

The text has been rewritten, also according to suggestions from Reviewer 1 (see above, top page 6 of the manuscript).

· Several figures report "percentage of flagellum" covered by a labeled protein (e.g. Fig 5A, B). The relevant question is what portions along the flagellum are labeled, so a more informative representation of the data would be to indicate what portion, base to tip, of the flagellum is labeled. Note that representative images should also be shown in suppl data

The presentation has been modified accordingly and the sentence: “The ratio between the length occupied by the indicated protein and the length of the axoneme is shown in percentage” has been added in the legend of the new figure 5 (pages 33-34). To facilitate comparison between control and teniposide-treated cells, we have shown graphs with the distribution of each component (outer dynein arms, distal and proximal docking complex) on the same figure (new figure 5) with panel A for untreated *lrrc56*$^{-/-}$ cells and panel B for teniposide-treated *lrrc56*$^{-/-}$ cells. We have added more representative images (in addition to those already shown in the new figure 5C-D) in the new Figure S4 as requested, with individual staining for dDC2, pDC1 and DNAI1.

· All charts should show actual data points, e.g. as in Fig 1 D and E, rather than simple box and whiskers plots shown for most/all other charts. Even something like a violin plot would be more informative and realistic representation of the data.

In agreement with the suggestion, we have changed the data representation using violin plots in Figures 1C, 3D, 5A-B and S3.

· Authors mention shorter flagella in the abstract, but I didn't see any measurements of flagellum length (only the ratio of new/old).

This observation was mentioned in our previous paper (Bonnefoy et al., AJHG2018) but without showing the data. A new supplementary figure (Figure S3A) has been added showing flagellum length in *lrrc56*$^{-/-}$ and control cells, which is now described in the text on page 12. The figure has been completed with the data of teniposide-treated cells, showing the absence of rescue of the length phenotype (blue violins) compared to wild-type controls (black violins, Figure S3B), as discussed on page 12.

· In Fig. 3 legend, they say 'A non-specific signal is also observed at the base of the flagella of tagged mNG::dDC2 cells, exactly as found on control cells (not shown)' but the control cells in Fig 2A don't seem to show this same non-specific signal.

It is true that a faint signal is observed between the nucleus and the kinetoplast of tagged mNG::dDC2 cells, even in control trypanosomes. Because of its positioning, it probably corresponds to the lysosome. However, the relatively strong flagellar signal observed in control cells means that this lysosome signal is barely visible, in contrast to *lrrc56*$^{-/-}$ cells where the flagellar signal is reduced by 2- to 4-fold (Figure 3D). A sentence has been added to the legend of Figure 3 (page 32).

Text is confusing or odd in some places and should be gone through thoroughly to check for inconsistencies. For example:

○ Fig 1C. It is hard to reconcile the "significantly slower" speed claimed for lrrc56 vs IFT81 (p12) based on the plot shown in figure 1c, as the data indicate nearly identical speed, albeit broader distribution in speed for IFT81. Why does fig 1c shown as box and whiskers, instead of all the data points as shown for 1d and 1e? Should show all the data points?

We have revised this graph (as also proposed by Reviewer 1) to now show separately IFT81, LRRC56 associated with IFT81 and LRRC56 trafficking alone, using a violin presentation that shows more clearly the differences (Fig. 1C).

○ Fig 2 and text on p15. Absence of DC1/2 in the very distal tip of the flagellum in lrrc56 mutants is evident. But, the claim that singal intensity is "signfificantly reduced" is not evident. Either provide quantitation to support the claim, or remove.

The difference in signal intensity has been quantified and is reported in figure 3D (2-fold reduction in old flagella and 4-fold in new flagella).

○ Fig 3: arrows are white or yellw, but text says white or red.

This has been corrected (Figure 3 and page 10).

○ Fig 3C and text on p16. Care should be taken in claiming the dDC2 in the flagellum 'remains' associated with the cytoskeleton. The statement implies all the dDC2 that gets there remains stably associated with the axoneme, but that's not what the data show. The data show dDC2 can be detected after detergent extraction, but it is not comparing before vs after extraction. Indeed, the signal in panel 3C looks much reduced compared to non-extracted cells shown in 3A.. Authors could assess this via western blot of detergent-solubliized vs detergent insoluble fractions Otherwise, change the statement to indicate some dDC2 remains on the axoneme without implying it all does

We could not perform a proper quantification by Western blot due to the lack of reactivity of the anti-mNG by Western in our hands. We, therefore, adapted the text consequently (page 10) as suggested by the reviewer: "Some mNG::dDC2 detected by IFA in *lrrc56*$^{-/-}$ cells remained associated with the cytoskeleton".

Also: for fig 3D, please specify in figure legend whether intensity analysis was done on detergent-extracted samples.

This is indeed cytoskeletons, as now mentioned in the legend (page 33).

○ Fig 5C/D is very confusing:

-labels at top indicate "pd" DC
-schematic indicates "p" DC
-figure legend says "expressing mNG::pDC1 and mNG::pDC1", which is redundant.

We thank the reviewer for having noticed these typos that have been corrected (also picked up by reviewer 1), it is of course pDC1 (the redundant bit of the legend has been corrected too on page 33).

What is being examined?

The co-localization of the proximal docking complex (via tdT::pDC1) and of the outer dynein arm component DNAI1 (intermediate chain also called IC78). The legend has been rewritten to be more explicit and the images transferred to figure 4 to allow direct comparison with the live images of the proximal docking complex and the colocalization with the outer dynein arm marker DNAI1 (Figure 4, legend page 33).

○ The suggestion on p3/4 that nuanced, species-specific differences in lrrc56 mutant defects is "unusual" seems a bit overstated. I think there are several examples of species-specific differences in phenotype for other flagellar proteins.

We agree that other differences in phenotypes have been reported for some flagellar proteins but we are discussing here only about outer dynein arms. This has been stated more clearly in the text to avoid ambiguity: “However, an unusual situation for <u>outer dynein arms</u>” (bottom page 4).

○ p4, near end of page: authors say LRRC56 is found in the flagellum MATRIX. Do they mean matrix, as in SOLUBLE, or do they mean to say axoneme? or is it both? Answering this would require authors to do comparison of LRRC56 distribution between supe and pellet from detergent-extracted cells. (this is relevant to comments elsewhere concerning how much of the lrrc56 protein remains associated with the axoneme upon detergent extraction) .

Due to the technical problem with the anti-mNG in western blots mentioned above, these quantifications could not be done. We therefore replaced the sentence with “was solubilised upon detergent treatment” (page 5).

○ Please explain the statement on p12 that LRRC56 is absent from mature flagella.

We showed previously that LRRC56 is abundant while the flagellum is being constructed but progressively disappears during maturation. We modified the sentence on page 8 to say; “as LRRC56 disappears during flagellum maturation as shown previously (Bonnefoy *et al.*, 2018)”.

○ P12, statement that Fig 1A,B provide the "first" evidence for lrrc56 as IFT cargo. This doesn't seem to be correct, because these authors showed previously that lrr56 co-localized with IFT particles, and I think some data have shown LRRC56 to coIP with IFT proteins. So, the current data show movement, but it's not the first evidence...

It is correct that LRRC56 can be co-immunoprecipitated with IFT88, but this experiment was done upon transfection of HEK293 cells with constructs expressing tagged versions of the two proteins. It showed their potential to associate but does not demonstrate that this occurs

during IFT since these cells were not ciliated in the experimental conditions used. Results obtained in this live system have now clearly answered this issue, explaining our statement.

○ In the 1st paragraph of results, authots say 'Acquisitions were performed on monoflagellated cells displaying an mNG::LRRC56 fluorescent signal, therefore corresponding to cells that inherited a new flagellum after cytokinesis, LRRC56 being absent from the mature flagellum.' I don't understand this comment - please clarify.

As said above, we showed previously that LRRC56 is abundant while the flagellum is being constructed but progressively disappears during maturation.

○ p. 24 'dramatic loss of mNG::dDC1 signal was observed': Are the authors saying that the cytoplasmic dDC1 signal is reduced in intensity compared to controls? It is not clear based on how they scaled their images whether overall signal is decreased or whether there is mislocalization to the cytoplasm. If they are referring to loss of flagellar signal, that should be clarified .

This is indeed the flagellar signal and is now clearly stated in the text: "dramatic loss of flagellar mNG::dDC1 signal" (page 13)

○ Fig S1: I'm not familiar with how one does 'background subtraction' on kymographs to get just the anterograde ones. Can the authors elaborate in the methods?

This is a routine procedure to extract the anterograde traces and improve signal-to-noise ratio, based on the Fourier transform (see Chenouard et al., 2010). One sentence has been added in the Method section: "… where the anterograde kymographs were separated from the whole kymographs to improve signal-to-noise ratio and analyzed as described (Chenouard et al., 2010; de Chaumont et al., 2012; Buisson et al., 2013)."

RE: Manuscript #E23-11-0425R
TITLE: "LRRC56 is an IFT cargo required for assembly of the distal dynein docking complex in Trypanosoma brucei"

Dear Dr. Bonnefoy:

The revision of your manuscript has been reviewed. Both reviewers thought that the manuscript has been improved. However, there are a number of remaining concerns from both reviewers. I concur with their comments. Please fully address these criticisms in your resubmission. No new experiments are needed, but revising the manuscript based on these concerns is necessary for better representation and more rigorous interpretation of the data. For example, currently, the expansion of pDC1 distribution in the lrrc56 mutant is interpreted as pDC1 "filling progressively the unoccupied distal docking sites". I agree with reviewer 2 that this interpretation is too narrow and simplistic, given that there is no biochemical data presented to show that pDC1 and dDC might share similar binding sites. Alternative models, such as a change in the kinetics of flagellar transport, can also explain the change in pDC1 distribution.

Please reassign the panel labelings in the figures according to MBoC style. Sub panels labelings (e.g. Fig X Aa, Ab etc..) within a figure panel are not allowed.

Please revise the manuscript based on the reviewers' critiques and provide a point-by-point response.

Sincerely,
Ke Hu
Monitoring Editor
Molecular Biology of the Cell

Dear Dr. Bonnefoy,

The review of your manuscript, referenced above, is now complete. The Monitoring Editor has decided that your manuscript requires minor revisions before it can be published in Molecular Biology of the Cell, as described in the Monitoring Editor's decision letter above and the reviewer comments (if any) below.

A reminder: Please do not contact the Monitoring Editor directly regarding your manuscript. If you have any questions regarding the review process or the decision, please contact the MBoC Editorial Office (mboc@ascb.org).

When submitting your revision include a rebuttal letter that details, point-by-point, how the Monitoring Editor's and reviewers' comments have been addressed. (The file type for this letter must be "rebuttal letter"; do not include your response to the Monitoring Editor and reviewers in a "cover letter.") Please bear in mind that your rebuttal letter will be published with your paper if it is accepted, unless you have opted out of publishing the review history.

Authors are allowed 15 days to submit a revision. If this time period is inadequate, please contact us immediately at mboc@ascb.org.

In preparing your revised manuscript, please follow the instruction in the Information for Authors (www.molbiolcell.org/info-for-authors). In particular, to prepare for the possible acceptance of your revised manuscript, submit final, publication-quality figures with your revision as described.

To submit the rebuttal letter, revised version, and figures, please use this link (please enable cookies, or cut and paste URL):
Link Not Available

MBoC offers the option to publish your work with immediate open access. Open access can increase the discoverability and usability of your research. If you would like to publish your paper with immediate open access but did not select this option during initial submission, please contact us at mbc@ascb.org..

Authors of Articles and Brief Communications whose manuscripts have returned for minor revision ("revise only") are encouraged to create a short video abstract to accompany their article when it is published. These video abstracts, known as Science Sketches, are up to 2 minutes long and will be published on YouTube and then embedded in the article abstract. Science Sketch Editors on the MBoC Editorial Board will provide guidance as you prepare your video. Information about how to prepare and submit a video abstract is available at www.molbiolcell.org/science-sketches. Please contact mboc@ascb.org if you are interested in creating a Science Sketch.

Thank you for submitting your manuscript to MBoC. Please do not hesitate to contact this office if you have any questions.

Sincerely,

Eric Baker
Journal Production Manager
MBoC Editorial Office
mbc@ascb.org

Reviewer #1 (Remarks to the Author):

Report on Bonnefoy et al. "Novel roles for the LRRC56 protein ....".

The authors have addressed most of my concerns in a satisfactory manner.

-> I noticed that the current manuscript has two different version of figure 5.

-> Some changes described or cited in the rebuttal letter are different from what is actually written in the manuscript.

-> I wonder if the authors could speculate what might drive the IFT-independent transport of LRRC56.

-> Fig. S1B is not convincing, as the overall pattern of fluorescence changes. This makes it unclear whether the same particle is marked in the frames. I suggest to mark the same particles in the kymogram shown in A panel b.

-> The form of the significance statement (with a heading and three bullet points) appears not to be journal style.

Reviewer #2 (Remarks to the Author):

• The paper is improved. However, my original main concern remains. Namely, that expansion of pDC1 distribution in the lrrc56 mutant is not simply reflecting pDC moving into sites no longer occupied by dDC2.
○ The authors argue that expanded distribution of pDC1 is simply due to pDC1 having access to sites that are no longer occupied by dDC. I do not think the data support that interpretation. For example, compare the distribution of dDC2 in fig 2B and that of pDC1 in fig 4B. The distribution of dDC2 and pDC1 look nearly identical (See attached Fig = panels from Fig 2B and 4B). The region where dDC2 is most lacking is at the extreme distal end of the flagellum (Fig 2B) and this region also lacks pDC1. A more nuanced model is suggested, e.g. one in which transport is altered, rather than simply binding, as the data indicate there is more going on here than simple competition for DC binding sites.
{section sign} The suggestion supplied by the authors in rebuttal, namely that the discrepancy is explained by "the resolution of optical microscopy" is not satisfactory.
{section sign} I agree that dDC does not extend as far to the extreme distal end of the flagellum in lrrc56 mutants vs WT, and that pDC extends more distal in the mutant vs WT. However, the suggestion that extension of pDC distribution simply reflects access to sites vacated by dDC is not supported, as the sites most vacated by dDC remain unoccupied by pDC. (If the authors wish to explore this further, experiments in which dDC and pDC location are examined directly in the same cells is needed.)
• QUESTION: Why is the image in the right-hand panel of Fig 2B repeated as Fig 3A? (If to contrast with detergent-extracted flagella in Fig 3B, why not simply refer back to Fig 2B instead of repeating the image? If the authors are making a different point than they made upon description of Fig 2B, they should simply refer back to Fig 2B)
• Fig 4A and 4B: please adjust the position of asterisks, as they do not align with the flagellum tip in all cases, and should be shown in all panels.

For the "Significance Statement", the term "functional genomics" is not appropriate, as genomics is not employed in the paper. "functional analysis" would be more applicable.

Manuscript #E23-11-0425R: point by point response letter

TITLE: Novel roles for the LRRC56 protein, an IFT cargo protein, in docking of dynein arms in *Trypanosoma brucei*

We thank the editor and the reviewers for their interest, support and constructive suggestions. Our responses are below in blue.

Response to editor suggestions

The revision of your manuscript has been reviewed. Both reviewers thought that the manuscript has been improved. However, there are a number of remaining concerns from both reviewers. I concur with their comments. Please fully address these criticisms in your resubmission. No new experiments are needed, but revising the manuscript based on these concerns is necessary for better representation and more rigorous interpretation of the data. For example, currently, the expansion of pDC1 distribution in the lrrc56 mutant is interpreted as pDC1 "filling progressively the unoccupied distal docking sites". I agree with reviewer 2 that this interpretation is too narrow and simplistic, given that there is no biochemical data presented to show that pDC1 and dDC might share similar binding sites. Alternative models, such as a change in the kinetics of flagellar transport, can also explain the change in pDC1 distribution.

We agree with the editor and reviewer 2 that this interpretation was too narrow and have developed the interpretation of results based on published data from Edwards et al., 2018 respective to the fate of the proximal docking complex upon distal docking complex knockdown (see response to reviewer 2). We propose an explanation as to the presence of the proximal docking complex beyond its distal counterpart, combining molecule availability and diffusion rather than transport processes.

Please reassign the panel labelings in the figures according to MBoC style. Sub panels labelings (e.g. Fig X Aa, Ab etc..) within a figure panel are not allowed.

The labeling of the sub-panels from Fig. 1 and Fig. S1 have been modified to fit MBoC style. Legends and text have been modified accordingly.

Please revise the manuscript based on the reviewers' critiques and provide a point-by-point response.

Reviewer #1 (Remarks to the Author):

Report on Bonnefoy et al. "Novel roles for the LRRC56 protein ....".

The authors have addressed most of my concerns in a satisfactory manner.

-> I noticed that the current manuscript has two different version of figure 5.

This was an error and has been corrected

-> Some changes described or cited in the rebuttal letter are different from what is actually written in the manuscript.

We apologize for these modifications done after rebuttal letter writing.

-> I wonder if the authors could speculate what might drive the IFT-independent transport of LRRC56.

We added the following paragraph at page 15

The pattern and relatively fast speed of this movement are unlikely to correspond to diffusion, and the precise mechanisms of these transport events of LRRC56, remain an open question. Apart from the kinesin-2 molecular motors responsible for anterograde transport (Bertiaux et al. 2018b), more than 40 kinesin-like proteins are encoded by the *T. brucei* genome (Berriman et al., 2005; Wickstead and Gull, 2006; Wickstead et al., 2010), several of them being located in the flagellum (Subota et al., 2014)(Billington et al., 2023) that might account for the independent transport of LRRC56.

-> Fig. S1B is not convincing, as the overall pattern of fluorescence changes. This makes it unclear whether the same particle is marked in the frames. I suggest to mark the same particles in the kymogram shown in A panel b.

We have marked the corresponding particle on the image of the kymograph (Figure S1, now panel D) as suggested.

-> The form of the significance statement (with a heading and three bullet points) appears not to be journal style.

We are proposing the following statements (page 3):

Loss or mutations of LRRC56, a protein that is conserved in organisms with motile cilia assembled by intraflagellar transport has variable impacts on outer dynein arms (ODA) from one species to the other.

Here, using functional analysis and live imaging, the authors demonstrate that LRRC56 is a cargo of intraflagellar transport in the protist *Trypanosoma brucei* and that interaction of LRRC56 with the docking complex is responsible for the absence of ODA.

Because LRRC56 is mutated in some patients suffering from ciliopathies, the results are of significance for both cell biology and physio-pathology.

Reviewer #2 (Remarks to the Author):

The paper is improved. However, my original main concern remains. Namely, that expansion of pDC1 distribution in the lrrc56 mutant is not simply reflecting pDC moving into sites no longer occupied by dDC2.

○ The authors argue that expanded distribution of pDC1 is simply due to pDC1 having access to sites that are no longer occupied by dDC. I do not think the data support that interpretation. For example, compare the distribution of dDC2 in fig 2B and that of pDC1 in fig 4B. The distribution of dDC2 and pDC1 look nearly identical (See attached Fig = panels from Fig 2B and 4B). The region where dDC2 is most lacking is at the extreme distal end of the flagellum (Fig 2B) and this region also lacks pDC1. A more nuanced model is suggested, e.g. one in which transport is altered, rather than simply binding, as the data indicate there is more going on here than simple competition for DC binding sites.

{section sign} The suggestion supplied by the authors in rebuttal, namely that the discrepancy is explained by "the resolution of optical microscopy" is not satisfactory.

{section sign} I agree that dDC does not extend as far to the extreme distal end of the flagellum in lrrc56 mutants vs WT, and that pDC extends more distal in the mutant vs WT. However, the suggestion that extension of pDC distribution simply reflects access to sites vacated by dDC is not supported, as the sites most vacated by dDC remain unoccupied by pDC. (If the authors wish to explore this further, experiments in which dDC and pDC location are examined directly in the same cells is needed.)

We agree that our discussion was too simplistic. We have revisited our data and we are now proposing an explanation taking into consideration the actual amount of proximal DC and its ability to diffuse and occupy the whole length of the axoneme. This proposal explains the difference we observed in terms of proximal DC spreading (more than 90% of the axoneme length) compared to published results from Edwards et al., 2018 who observed a maximum of 75% occupancy by the proximal DC in total absence of distal DC.

This is mentioned in the results (page 11-13) and explained extensively in the discussion at page 16:

Edwards *et al.*, 2018 have proposed that retrograde transport of proximal DCs generates a concentration gradient in which the higher-affinity proximal DC is restricted to the proximal axoneme, while the lower-affinity and unbound distal DC would diffuse throughout the flagellum and fill the remaining axoneme binding sites. In absence of distal complex proteins, those of the proximal docking complex progressively occupy the space left vacant and ensure proper attachment of the dynein arms. Nevertheless, they are restricted to ~75% of the flagellum length (Edwards et al., 2018). Here, in absence of LRRC56 protein, proximal DC components can reach more than 90% of flagellum length (Fig. 5) either in old flagella or in new flagella of cells treated for 20h with teniposide. We propose that the available amount of proximal DC components is only 75% of what would be required to occupy the full length of

the axoneme, explaining the published results (Edwards et al., 2018). However, in *lrrc56*$^{-/-}$ cells, a low amount of distal DC proteins are still present in the middle portion of the axoneme, spreading over ~50% of the length but with a 2-3 fold-lower density compared to controls (Fig. 3C), suggesting that ~20-25% of the binding sites on axonemal microtubules are occupied by the distal DC. In these conditions, the amount of proximal DC would be sufficient to diffuse slowly and to progressively fill in the rest of the axoneme, explaining the >90% occupancy observed in old flagella or in new flagella of cells treated with teniposide.

• QUESTION: Why is the image in the right-hand panel of Fig 2B repeated as Fig 3A? (If to contrast with detergent-extracted flagella in Fig 3B, why not simply refer back to Fig 2B instead of repeating the image? If the authors are making a different point than they made upon description of Fig 2B, they should simply refer back to Fig 2B).

We have replaced Fig. 2B right panel with former 3A that contains the complete set of annotations. This has been removed from figure 3 as requested and text has been modified accordingly.

• Fig 4A and 4B: please adjust the position of asterisks, as they do not align with the flagellum tip in all cases, and should be shown in all panels.

Fig 4A and 4B have been corrected.

For the "Significance Statement", the term "functional genomics" is not appropriate, as genomics is not employed in the paper. "functional analysis" would be more applicable.

In the new statement, this term was modified as requested.

RE: Manuscript #E23-11-0425RR
TITLE: "LRRC56 is an IFT cargo required for assembly of the distal dynein docking complex in Trypanosoma brucei"

Dear Dr. Bonnefoy:

Thanks for resubmitting your manuscript. The revision has addressed most of the issues raised in the last round of review. However, in the rebuttal letter, Figure 3A from the last submission is said to have been removed, but this panel (identical to Figure 2B, right panel) still remains in the current Figure 3 even though the main text has been edited based on the version described in the rebuttal letter. This type of inconsistencies has been a recurring problem during the review process. It generates confusion and does not reflect favorably on the rigor of the preparation of the manuscript. Additionally, while Figure S1D has been updated to address Reviewer 1's comment, the notations are still unclear. The magenta and white lines are supposed to be the traces of the particles indicated by arrowheads in Figure S1E. However, in Figure S1E, the magenta, green, and white arrowheads are aligned in all frames starting from time 0, but in Figure S1D the white line only partially overlap with the magenta line. Please revise the text and the figure to clarify what exactly the arrowheads and traces represent.

Please make the appropriate corrections in the next revision. This will be the final chance for this manuscript to be considered for publication.

Sincerely,
Ke Hu
Monitoring Editor
Molecular Biology of the Cell

PS: Please revise your Significance Statement so it consists of three separate bullet points totaling a maximum of 100 words and addressing each of the following points (see https://www.molbiolcell.org/curation-tools):
First bullet: What is the background context? What gap in knowledge does this study addres?
Second bullet: What are the key findings? What is unique or new about the approach?
Third bullet: Why is this paper significant? How might it influence future research?

Dear Dr. Bonnefoy,

The review of your manuscript, referenced above, is now complete. The Monitoring Editor has decided that your manuscript requires minor revisions before it can be published in Molecular Biology of the Cell, as described in the Monitoring Editor's decision letter above and the reviewer comments (if any) below.

A reminder: Please do not contact the Monitoring Editor directly regarding your manuscript. If you have any questions regarding the review process or the decision, please contact the MBoC Editorial Office (mboc@ascb.org).

When submitting your revision include a rebuttal letter that details, point-by-point, how the Monitoring Editor's and reviewers' comments have been addressed. (The file type for this letter must be "rebuttal letter"; do not include your response to the Monitoring Editor and reviewers in a "cover letter.") Please bear in mind that your rebuttal letter will be published with your paper if it is accepted, unless you have opted out of publishing the review history.

Authors are allowed 15 days to submit a revision. If this time period is inadequate, please contact us immediately at mboc@ascb.org.

In preparing your revised manuscript, please follow the instruction in the Information for Authors (www.molbiolcell.org/info-for-authors). In particular, to prepare for the possible acceptance of your revised manuscript, submit final, publication-quality figures with your revision as described.

To submit the rebuttal letter, revised version, and figures, please use this link (please enable cookies, or cut and paste URL):
Link Not Available

MBoC offers the option to publish your work with immediate open access. Open access can increase the discoverability and usability of your research. If you would like to publish your paper with immediate open access but did not select this option during initial submission, please contact us at mbc@ascb.org..

Authors of Articles and Brief Communications whose manuscripts have returned for minor revision ("revise only") are

encouraged to create a short video abstract to accompany their article when it is published. These video abstracts, known as Science Sketches, are up to 2 minutes long and will be published on YouTube and then embedded in the article abstract. Science Sketch Editors on the MBoC Editorial Board will provide guidance as you prepare your video. Information about how to prepare and submit a video abstract is available at www.molbiolcell.org/science-sketches. Please contact mboc@ascb.org if you are interested in creating a Science Sketch.

Thank you for submitting your manuscript to MBoC. Please do not hesitate to contact this office if you have any questions.

Sincerely,

Eric Baker
Journal Production Manager
MBoC Editorial Office
mbc@ascb.org

Dear Editor, Dear Dr. Hu,

Many thanks for your message and for having identified the hiccups. We sincerely apologise for these errors and have modified the manuscript to correct them and to clarify the remaining point about kymograph annotations (see below).

Please accept our apologies and we hope that this revised version will be suitable for publication in Molecular Biology of the Cell.

Best regards,

Serge Bonnefoy, Aline Alves, Philippe Bastin

Response letter

Our responses are indicated in blue.

Thanks for resubmitting your manuscript. The revision has addressed most of the issues raised in the last round of review. However, in the rebuttal letter, Figure 3A from the last submission is said to have been removed, but this panel (identical to Figure 2B, right panel) still remains in the current Figure 3 even though the main text has been edited based on the version described in the rebuttal letter. This type of inconsistencies has been a recurring problem during the review process. It generates confusion and does not reflect favorably on the rigor of the preparation of the manuscript.

The corrected version of Figure 3 exactly matching the legend has now been submitted.

Additionally, while Figure S1D has been updated to address Reviewer 1's comment, the notations are still unclear. The magenta and white lines are supposed to be the traces of the particles indicated by arrowheads in Figure S1E. However, in Figure S1E, the magenta, green, and white arrowheads are aligned in all frames starting from time 0, but in Figure S1D the white line only partially overlap with the magenta line. Please revise the text and the figure to clarify what exactly the arrowheads and traces represent.

Thank you for having spotted this inconsistency. The exact time of each image frame is now indicated on the updated panel S1G. To facilitate reading and avoid any confusion, panels have been numbered individually and are now referred as such in the text of the manuscript (see text pages 8-9). The legends of both Figure 1 (page 25) and Figure S1 (page 29) have been updated accordingly.

Please revise your Significance Statement so it consists of three separate bullet points totaling a maximum of 100 words and addressing each of the following points (see https://www.molbiolcell.org/curation-tools):

First bullet: What is the background context? What gap in knowledge does this study addres?
Second bullet: What are the key findings? What is unique or new about the approach?
Third bullet: Why is this paper significant? How might it influence future research?

The significance statement has been modified as follows:

- We aimed to explain why absence of LRRC56, a protein conserved in organisms with motile cilia assembled by intraflagellar transport (IFT), leads to loss of outer dynein arms (ODA) only in the distal portion of the *Trypanosoma brucei* flagellum.

- We used functional analysis and live imaging to demonstrate that LRRC56 is an IFT cargo and that the interaction of LRRC56 with the distal docking complex is responsible for the absence of ODA.

- This work revealed how LRRC56 is transported and unveiled an unexpected link with the dynein docking complex, which might explain some phenotypes in ciliopathy patients with LRRC56 mutations.

RE: Manuscript #E23-11-0425RRR
TITLE: "LRRC56 is an IFT cargo required for assembly of the distal dynein docking complex in Trypanosoma brucei"

Dear Dr. Bonnefoy:

I am pleased to accept your manuscript for publication in Molecular Biology of the Cell.

Congratulations.

Sincerely,
Ke Hu
Monitoring Editor
Molecular Biology of the Cell

Dear Dr. Bonnefoy:

Congratulations on the acceptance of your manuscript! Thank you for choosing to publish your work in Molecular Biology of the Cell (MBoC).

Within 10 days, an unedited PDF of your manuscript will be published on MBoC in Press, an early release version of the journal. The date your manuscript appears on this site is the official publication date.

Your copyedited and typeset manuscript will be scheduled for publication in the next available issue of MBoC. Our production team will notify you when the page proofs of your paper are ready for your review.

MBoC offers the option to publish your paper with immediate open access. Open access can increase the discoverability and usability of your research. If you would like to publish your paper with immediate open access but did not select this option during initial submission, please contact the MBoC Editorial Office (mbc@ascb.org).

We invite you to submit images related to your accepted manuscript to be considered for the journal cover.
Cover images must contain color, TIF Format
Size image at 7.4" wide x 5.85" deep
RGB and CMYK color (please submit two separate files, one in each format)
300 dpi resolution

Please also send a 1-paragraph description of the image that a general audience could understand. Include with the caption the name and institution of the individual(s) the image should be attributed to. Please provide this description in a Word file. You can send the file via email or by your favorite file transfer service to mboc@ascb.org.

Authors of Articles and Brief Communications are also encouraged to create a short video abstract to accompany their article when it is published. These video abstracts, known as Science Sketches, are up to 2 minutes long and will be published on YouTube and then embedded in the article abstract. Science Sketch Editors on the MBoC Editorial Board will guide you as you prepare your video. Information about how to prepare and submit a video abstract is available at www.molbiolcell.org/science-sketches. Please contact mboc@ascb.org if you are interested in creating a Science Sketch.

We look forward to publishing your paper in MBoC.
Sign up for MBoC's new content alerts here https://tinyurl.com/3bcsyk3e

Sincerely,

Eric Baker
Journal Production Manager
MBoC Editorial Office
mbc@ascb.org
